# MGCG regulates glioblastoma tumorigenicity via hnRNPK/ATG2A and promotes autophagy

**DOI:** 10.1038/s41419-023-05959-x

**Published:** 2023-07-17

**Authors:** Fang Chu, Pengfei Wu, Maolin Mu, Shanshan Hu, Chaoshi Niu

**Affiliations:** 1 Department of Neurosurgery, The First Affiliated Hospital of USTC, Division of Life Sciences and Medicine, University of Science and Technology of China, Hefei, Anhui 230001 P.R. China; 2 Anhui Key Laboratory of Brain Function and Diseases, Hefei, Anhui 230001 P.R. China; 3 Anhui Provincial Stereotactic Neurosurgical Institute, Hefei, Anhui 230001 P.R. China; 4 Anhui Provincial Clinical Research Center for Neurosurgical Disease, Hefei, Anhui 230001 P.R. China

**Keywords:** CNS cancer, Macroautophagy

## Abstract

Glioblastoma (GBM) is the most common malignant primary brain cancer in adults and has constantly been a focus of research. Long noncoding RNAs (lncRNAs) play important roles in the development of cancers. To illustrate the role of lncRNAs in the development of glioblastoma, high-throughput RNA sequencing was performed to obtain the transcripts using three freshly isolated tumor tissue samples from GBM patients and three normal brain tissue samples from the traumatic brain of patients. Then, a lncRNA, MGCG (MGC70870 is expressed at a high level in glioblastoma), which has not been reported previously in GBM, was found to be associated with the prognosis of patients. The results of bioinformatic analysis showed that MGCG was correlated with autophagy and positively correlated with the expression of the autophagy-related gene ATG2A. The data of mass spectrometry demonstrated that the hnRNPK protein was a direct target interacting with MGCG, and MGCG/hnRNPK promoted the development of GBM by enhancing the translation of ATG2A and autophagy. In conclusion, the present study showed that MGCG has the potential to promote the development of GBM and may become a candidate for molecular diagnostics and treatment of tumors.

## Introduction

Adult glioblastoma (GBM) is one of the deadliest and most recalcitrant malignant solid tumors [1]. According to the pathological classification of the World Health Organization (WHO), GBM is classified into numerical grades (I-IV) [2]. GBM is considered incurable, with a median survival of 15 months following aggressive combination therapies, including maximal-safe surgical resection and adjuvant radiation therapy (RT) with concurrent adjuvant temozolomide (TMZ) treatment [3]. Currently, standard magnetic resonance imaging provides the most sensitive tool for the initial detection of GBM. However, when a GBM-definable lesion is identified by imaging, the tumor is in an advanced state [1]. Thus, molecular diagnosis, especially various biomarkers, must be investigated during GBM occurrence and progression.

Noncoding RNAs (ncRNAs) are common RNAs that do not encode a protein; however, this does not mean that such RNAs do not contain information or do not have a function [4]. Noncoding RNAs include microRNAs (miRNAs), lncRNAs, and circular RNAs (circRNAs) [5]. Many studies have demonstrated that lncRNAs play a significant regulatory role in shaping cellular activity and participate in biological processes in tumors [6–14]. For example, lncRNA NEAT1 has been demonstrated to bind to EZH2 and mediate the trimethylation of H3K27 in the promoter regions, thus promoting GBM cell growth and invasion [15]. LncRNA lnc-TALC traps miR-20b-3p, activates c-Met, increases MGMT expression, and may serve as a therapeutic target to overcome TMZ resistance [16]. All these findings show that lncRNAs play a significant role in the development and drug resistance of GBM. Thus, clinical significance and mechanisms underlying the effects of lncRNAs, especially in GBM, need to be further explored.

Autophagy is the major intracellular degradation system by which cytoplasmic materials are delivered to and degraded in the lysosome [17, 18]. However, simple elimination of the materials is not the purpose of autophagy; instead, autophagy serves as a dynamic recycling system that produces new building blocks and energy for cellular renovation and homeostasis [18]. The role of autophagy has been specifically emphasized in the development of cancer; once the tumor is diagnosed; increased autophagic flux often enables the survival and growth of the tumor cells [19, 20]. Autophagy is initiated and tightly regulated by a group of autophagy-related genes (ATGs) [21]. Many methods are used to manipulate autophagy by regulating ATGs for the treatment of cancer. However, the dysregulation of ATGs and their upstream regulators in GBM remains incompletely defined.

DNA methylation is an epigenetic mechanism involving the transfer of a methyl group onto the C5 position of the cytosine to form 5-methylcytosine [22]. DNA methylation is mainly catalyzed by three enzymes, DNMT1, DNMT3A, and DNMT3B [23]. Because DNA methylation represents a molecular mechanism associated with gene repression, it has been assumed that the modification in cancer may contribute to the tumor phenotype by inhibiting genes expression [24]. For example, DNA methylation-mediated lncRNA SNHG12 promotes temozolomide resistance in glioblastoma. Moreover, the roles of DNA methylation in GBM deserve a further study.

In the present study, we investigated the expression profile of lncRNAs using RNA-seq and identified an lncRNA upregulated in GBM, MGC70870, which we renamed as MGCG. Higher MGCG expression was related to poor prognosis of GBM patients. Furthermore, we demonstrated that MGCG influenced proliferation, migration, and apoptosis in vitro and inhibited tumor formation in vivo. In addition, the present study revealed a mechanism by which lncRNAs can upregulate the expression of ATG2A by combining with hnRNPK, thus promoting autophagy and tumor growth. The results of the present study indicated that MGCG acts as a tumor promoter in GBM and has a potential to be a promising target for diagnosis, therapy, and prognosis of GBM.

## Results

### MGCG is expressed at a high level in GBM and is correlated with poor prognosis

**Fig. 1:**
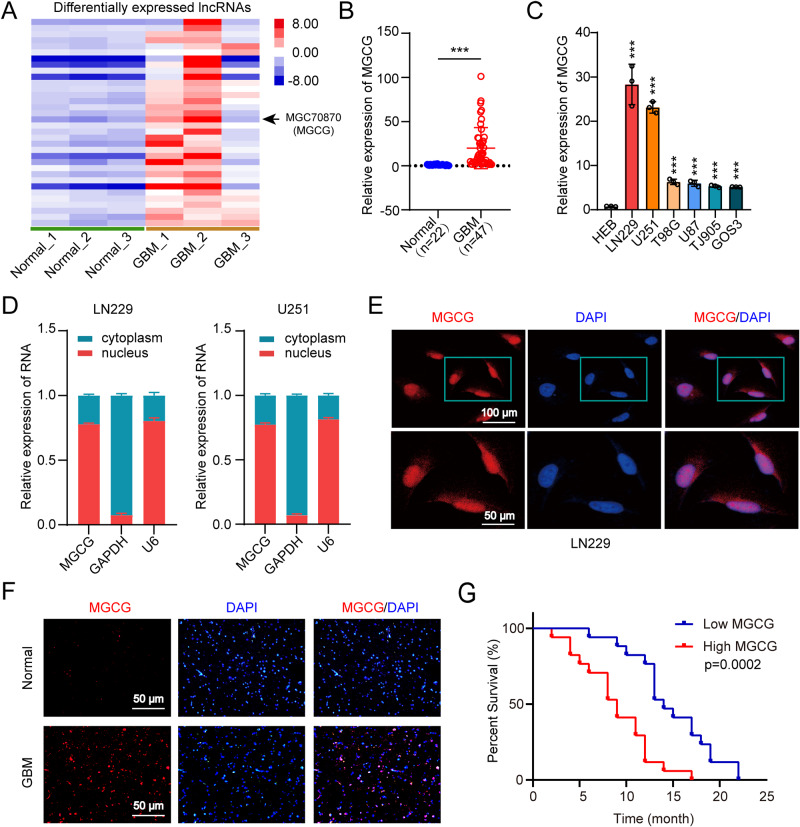
MGCG is overexpressed in GBM and leads to poor prognosis. **A** Heatmap showing the differential expression of MGCG in the normal and GBM tissues. **B** The expression of MGCG was assessed in the normal and GBM tissues by RT‒qPCR (normal *n* = 22, GBM *n* = 47). **C** RT‒qPCR analysis of the relative expression of MGCG in HEB and GBM cell lines. (GBM cell lines: LN229, U251, T98G, U87, TJ905, and GOS3). **D** RT‒qPCR analysis of MGCG expression in the nuclear and cytoplasmic fractions of LN229 and U251 cells. **E** FISH analysis of MGCG expression in the nuclear and cytoplasmic fractions of LN229 cells. **F** FISH analysis of the relative expression of MGCG in the paraffin sections of the normal GBM tissues. **G** Kaplan‒Meier plots of overall survival of clinical patients with GBM with high and low levels of MGCG. Error bars, SEM from three independent experiments. *P < 0.05; **P < 0.01; ***P < 0.001 by two-tailed Student’s test.

To obtain the expression profiles of lncRNAs and mRNAs, three GBM tissue samples (diagnosed by pathological biopsy) and three normal brain tissue samples from craniocerebral trauma patients were obtained, and high-throughput RNA sequencing was performed (GSE153692). The results of RNA sequencing showed that MGCG expression was significantly elevated in the GBM tissue compared with that in the normal tissue (Fig. 1A). Next, we verified the expression of MGCG in 22 normal brain tissue samples (craniocerebral trauma patients) and 47 GBM tissue samples by RT-qPCR and demonstrated that MGCG was expressed at significantly higher levels in the GBM tissue (Fig. 1B, Table 1). The results of RT-qPCR showed that the expression of MGCG was significantly higher in GBM cells than that in HEB cells (Fig. 1C). To identify cellular localization of MGCG, RT-qPCR of nuclear and cytoplasmic RNAs was carried out; the data showed that MGCG was preferentially located in the nucleus (Fig. 1D). Next, we performed RNA FISH to confirm these results in LN229 cells (Fig. 1E). The results of RNA FISH demonstrated that MGCG was upregulated in GBM versus normal tissue (Fig. 1F). We assessed a correlation between MGCG expression and the prognosis of GBM patients. The results of Kaplan–Meier analysis and correlation analysis between MGCG expression and clinical patient survival demonstrated that patients with higher MGCG expression had poor overall survival (Fig. 1G, Supplementary Fig. 1A). Overall, these data suggested that MGCG upregulation was common in the GBM tissue and was correlated with poor prognosis.

**Table 1:** The relationship of MGCG and clinical characteristics in 47 glioma patients.

Variable		MGCG		*p* value
		High (*n* = 24)	Low (*n* = 23)	
Sex				0.899
	Male	12	8	
	Female	12	15	
Age(year)				0.859
	≤55	14	13	
	>55	10	10	

### MGCG promotes tumor progression and autophagy in GBM

**Fig. 2:**
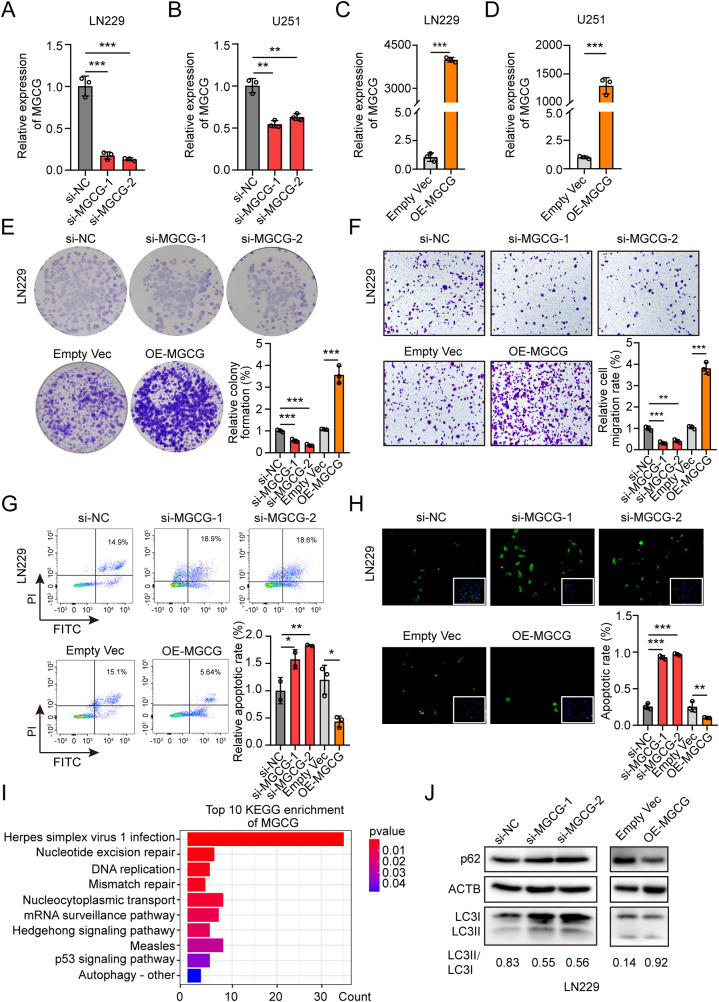
MGCG promotes malignant properties of GBM progression and induces autophagy. **A** The data of RT‒qPCR on the expression of MGCG in LN229 cells treated with si-NC and three independent siRNAs (si-MGCG1, si-MGCG2, and si-MGCG3). **B** The data of RT‒qPCR on the expression of MGCG in U251 cells treated with si-NC and three independent siRNAs (si-MGCG1, si-MGCG2, and si-MGCG3). **C** The data of RT‒qPCR on the expression of MGCG in LN229 cells treated with EMPTY VEC and OE-MGCG. **D** The data of RT‒qPCR on the expression of MGCG in U251 cells treated with EMPTY VEC and OE-MGCG. **E** Colony formation assays were used to detect the effect of MGCG knockdown or overexpression on the growth of LN229 cells. **F** Transwell assay shows the effects of MGCG knockdown or overexpression on the migration of LN229 cells. **G** The data of flow cytometry analysis revealed the effect of MGCG knockdown or overexpression on apoptosis of LN229 cells. **H** The data of TUNEL analysis demonstrate the effect of MGCG knockdown or overexpression on apoptosis of LN229 cells. **I** Top 10 KEGG pathway enrichment of MGCG. **J** Western blot analysis of the protein levels of p62 and LC3 after knockdown or overexpression of MGCG in LN229 cells. Error bars, SEM from three independent experiments. *P < 0.05; **P < 0.01; ***P < 0.001 by two-tailed Student’s test.

To explore the biological functions of MGCG, three independent small interfering RNAs (siRNAs) against MGCG were designed, and we selected two siRNAs that effectively knocked down the expression of MGCG in LN229 and U251 cells (Fig. 2A, B). We also constructed an overexpression plasmid and successfully overexpressed MGCG in LN229 and U251 cells (Fig. 2C, D). The results of the colony formation assays demonstrated that depletion of MGCG markedly inhibited cell proliferation, whereas overexpression of MGCG facilitated cell viability (Fig. 2E, Supplementary Fig. 1B). We confirmed that downregulation of MGCG significantly reduced the migratory capacity of the cells in the Transwell assays; however, MGCG overexpression increased the migratory capacity of the cells (Fig. 2F, Supplementary Fig. 1C). In addition, the results of the flow cytometry and TUNEL assays demonstrated that knockdown of MGCG induced cellular apoptosis and upregulated expression of MGCG inhibited cellular apoptosis (Fig. 2G, H, Supplementary Fig. 1D, E).

The role of MGCG in GBM was investigated based on the RNA sequencing data; we performed bioinformatic analysis and demonstrated that MGCG was associated with autophagy (Fig. 2I). The results of western blotting showed that MGCG overexpression increased LC3-II conjugation and p62 degradation, suggesting that MGCG induced autophagy in GBM cells (Fig. 2J, Supplementary Fig. 1F). Meanwhile, we observed the decreased number of autophagosomes when downregulation of MGCG and the increased number of autophagosomes when overexpressed MGCG in LN229 and U251 cells (Supplementary Fig. 2A, B). Moreover, the MDC stain (monodansylcadaverin, used as a specific marker stain to detect autophagosomes formation) and flow cytometry demonstrated that knockdown MGCG significantly reduced the number of autophagosomes, whereas overexpression of MGCG induced autophagy in GBM cells (Supplementary Fig. 2C, D). Chloroquine (CQ) is a recognized autophagy inhibitor, we treated GBM cells with CQ, and found CQ also inhibited autophagy in GBM cells by GFP-LC3 adenovirus infected GBM cells and flow cytometry (Supplementary Fig. 2E, F). The results indicated that the expression of MGCG was associated with malignant phenotype of GBM and that MGCG promoted autophagy in GBM.

### MGCG promotes GBM progression by regulating the expression of ATG2A

**Fig. 3:**
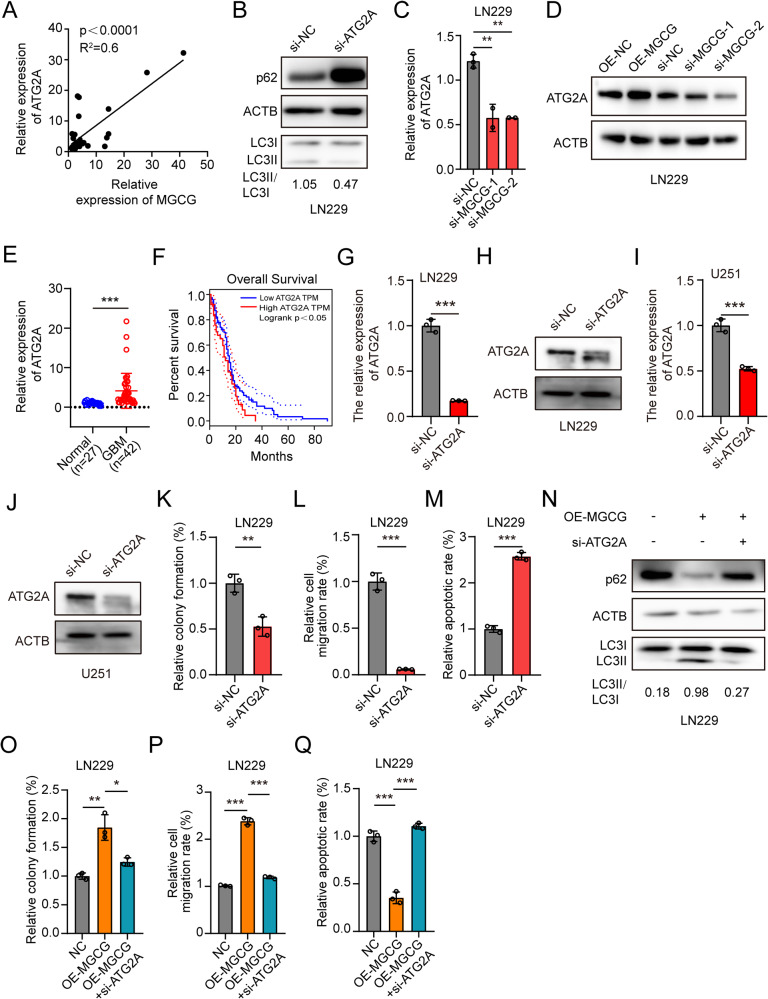
MGCG facilitates GBM progression by promoting the expression of ATG2A in LN229 cells. **A** RT‒qPCR analysis of the relative expression of MGCG and ATG2A mRNA in GBM tissue samples. **B** Western blot analysis was used to detect the protein levels of p62 and LC3 after ATG2A knockdown in LN229 cells. **C** RT‒qPCR analysis of the relative expression of ATG2A after MGCG knockdown in LN229 cells. **D** Western blot analysis of the protein levels of ATG2A after MGCG overexpression or knockdown in LN229 cells. **E** The expression of ATG2A was assayed in the normal and GBM tissues by RT‒qPCR (normal *n* = 27, GBM *n* = 42). **F** Kaplan‒Meier plots of overall survival of GBM patients with high and low levels of ATG2A. **G** The data of RT‒qPCR on the expression of ATG2A in LN229 cells treated with si-NC and si-ATG2A. **H** Western blot was used to detect the ATG2A protein level after treated with si-NC and si-ATG2A in LN229 cells. **I** The data of RT‒qPCR on the expression of ATG2A in U251 cells treated with si-NC and si-ATG2A. **J** Western blot was used to detect the ATG2A protein level after treated with si-NC and si-ATG2A in U251 cells. **K** Colony formation assays were used to detect the effect of ATG2A knockdown on the growth of LN229 cells. **L** The data of Transwell analysis show the effect of ATG2A knockdown on the migration of LN229 cells. **M** The data of TUNEL analysis show the effect of ATG2A knockdown on apoptosis of LN229 cells. **N** Western blot analysis of the protein levels of p62 and LC3 after MGCG overexpression or overexpression and simultaneous knockdown of ATG2A. **O** Colony formation assays were used to detect the effect of MGCG overexpression or overexpression and simultaneous knockdown of ATG2A on the growth of LN229 cells. **P** The data of Transwell analysis show the effect of MGCG overexpression or overexpression of MGCG and simultaneous knockdown of ATG2A on the migration of LN229 cells. **Q** The data of TUNEL analysis show the effect of MGCG overexpression or overexpression and simultaneous knockdown of ATG2A on apoptosis of LN229 cells. Error bars, SEM from three independent experiments. *P < 0.05; **P < 0.01; ***P < 0.001 by two-tailed Student’s test.

Many studies have demonstrated that autophagy promotes GBM development. Consistently, we demonstrated that autophagy was elevated in GBM cells overexpressing MGCG. ATG2A is an autophagy-related gene, and we already know that knockdown ATG2A inhibits autophagy [25], ATG2A has been seldom studied. The data of RT-qPCR proved that the expression of MGCG was significantly positively correlated with ATG2A in the clinical samples of the GBM tissue (Fig. 3A). P62 is the substrate of autophagy, and the expression of p62 is downregulated during autophagy. At the same time, the ratio of LC3-II to LC3-I increased. Next, we knocked down ATG2A and demonstrated that the ratio of the protein levels of LC3-II to LC3-I was decreased and the protein levels of p62 were increased in LN229 cells (Fig. 3B, Supplementary Fig. 2A). To investigate whether MGCG regulates the expression of ATG2A, we knocked down MGCG and demonstrated that the expression levels of ATG2A mRNA and protein were decreased according to the data of RT-qPCR and western blotting, respectively (Fig. 3C, D, Supplementary Fig. 2B, C). We verified the expression of ATG2A in 27 normal tissue samples and 42 GBM tissue samples by RT-qPCR and demonstrated that ATG2A was expressed at significantly higher levels in the GBM tissue (Fig. 3E). Moreover, lower expression of ATG2A was associated with improved overall survival of patients according to the TCGA database (Fig. 3F). In addition, we studied the effect of ATG2A on GBM progression. Firstly, we performed RT-qPCR and western blot to detect the knockdown efficiency of ATG2A in GBM cells (Fig. 3G–J). Downregulation of ATG2A inhibited the proliferation (Fig. 3K, Supplementary Fig. 3D) and migration of GBM cells (Fig. 3L, Supplementary Fig. 3E) and promoted apoptosis of GBM cells (Fig. 3M, Supplementary Fig. 3F). Then, ATG2A siRNA-containing and MGCG-overexpressing plasmids were co-transfected into LN229 and U251 cells, and we demonstrated that downregulation of ATG2A inhibited autophagy and that overexpression of MGCG rescued the inhibition of autophagy (Fig. 3N, Supplementary Fig. 3G). Knockdown of ATG2A abolished the effect of MGCG overexpression on the proliferation, migration, and apoptosis of the cells (Fig. 3O–Q, Supplementary Fig. 3H–J). All these results showed that MGCG regulated the expression of ATG2A and promoted autophagy.

### MGCG directly interacts with hnRNPK and facilitates ATG2A translation

**Fig. 4:**
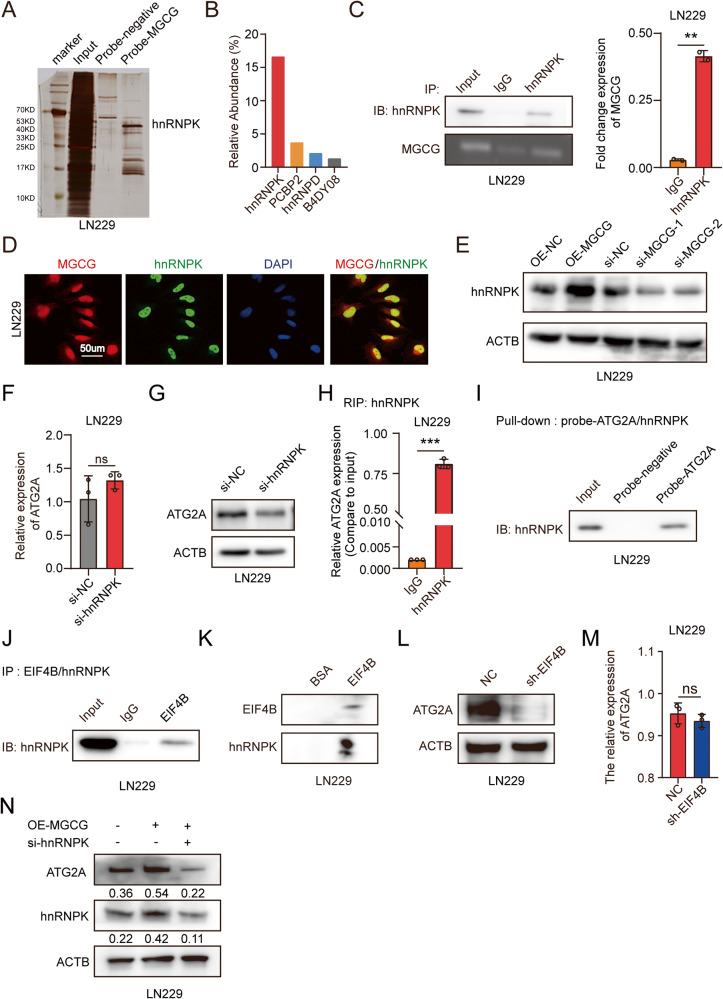
MGCG binds to hnRNPK and promotes ATG2A translation in GBM. **A** Silver staining of RNA pulldown from GBM cells expressing the biotin-tagged negative and MGCG probes. **B** Relative abundance of the proteins bound to MGCG determined by mass spectrometry. **C** RNA immunoprecipitation (RIP) with an anti-hnRNPK antibody in GBM cells. Left: MGCG enrichment was detected by nucleic acid gel electrophoresis. Right: Statistical evaluation of the abundance of IgG and MGCG assayed as shown in the left panel. **D** FISH and immunofluorescence (IF) were performed to show that MGCG colocalizes with hnRNPK in LN229 cells. **E** Western blot analysis of the protein levels of hnRNPK after MGCG overexpression or knockdown in LN229 cells. **F** RT‒qPCR analysis of the relative expression of ATG2A after hnRNPK knockdown in LN229 cells. **G** Western blot analysis of the protein levels of ATG2A after hnRNPK knockdown in LN229 cells. **H** RIP was performed with an anti-hnRNPK antibody in GBM cells, and the enrichment of ATG2A was detected by RT‒qPCR. **I** RNA pulldown was performed using GBM cells expressing the biotin-tagged negative and ATG2A probes, and the expression of hnRNPK was detected by western blotting. **J** Immunoprecipitation (IP) was performed using LN229 cells with an anti-EIF4B antibody, and the expression of hnRNPK was detected by western blotting. **K** Far western blot was used to detect the interaction between hnRNPK and EIF4B with EIF4B and hnRNPK recombinant protein. **L** Western blot analysis of the protein levels of ATG2A after EIF4B knockdown in LN229 cells. **M** RT‒qPCR analysis of the relative expression of ATG2A after EIF4B knockdown in LN229 cells. **N** Western blot analysis of the expression of hnRNPK with MGCG overexpression or overexpression of MGCG and simultaneous knockdown of hnRNPK in LN229 cells. Error bars, SEM from three independent experiments. *P < 0.05; **P < 0.01; ***P < 0.001 by two-tailed Student’s test.

To further explore the mechanism of action of MGCG in GBM, we conducted an RNA pulldown assay using a specific biotin-labeled MGCG probe. The results of silver staining showed several additional bands of proteins compared with the bands detected in the case of a negative probe, and mass spectrometry analysis identified hnRNPK, which is one of the major pre-mRNA-binding proteins, as the major protein that was bound to MGCG (Fig. 4A, B). The data of a RIP assay confirmed direct interaction between MGCG and hnRNPK (Fig. 4C). Consistently, the data of FISH and IF showed that MGCG and hnRNPK were colocalized in GBM cells (Fig. 4D). The results of western blot analysis showed that knockdown of MGCG or overexpression of MGCG inhibited or elevated, respectively, the protein levels of hnRNPK in GBM cells (Fig. 4E). To determine whether hnRNPK can regulate ATG2A expression, we performed RT-qPCR and demonstrated the lack of the changes in the RNA levels of ATG2A upon hnRNPK knockdown (Fig. 4F); the protein levels of ATG2A were downregulated when hnRNPK was knocked down, as shown by western blotting (Fig. 4G). Numerous studies have demonstrated that hnRNPK regulates translation [26]. To determine whether hnRNPK regulates ATG2A translation in GBM, we used RIP to demonstrate that hnRNPK was bound to ATG2A mRNA (Fig. 4H), and the results of RNA pulldown demonstrated an interaction between hnRNPK and ATG2A mRNA (Fig. 4I). HnRNPK regulates translation by binding a translation initiation factor and EIF4B is one of the translation initiation factor has been reported bind to hnRNPK [27]. We performed IP and confirmed that hnRNPK was bound to EIF4B in GBM (Fig. 4J). Far western blot had been reported to detect the protein-protein interactions [28]. We further confirmed the interaction between hnRNPK and EIF4B by far western blot (Fig. 4K). The results of western blot showed that knockdown of EIF4B inhibited the protein levels of ATG2A; however, there was no change in the RNA levels of ATG2A after downregulated EIF4B in LN229 cells (Figs. 4L, M). Knockdown of hnRNPK decreased the protein levels of ATG2A; however, overexpression of MGCG abolished the effect of hnRNPK knockdown (Fig. 4N). These results showed that MGCG facilitated the translation of ATG2A by binding hnRNPK.

### DNA hypomethylation upregulates MGCG expression in GBM

**Fig. 5:**
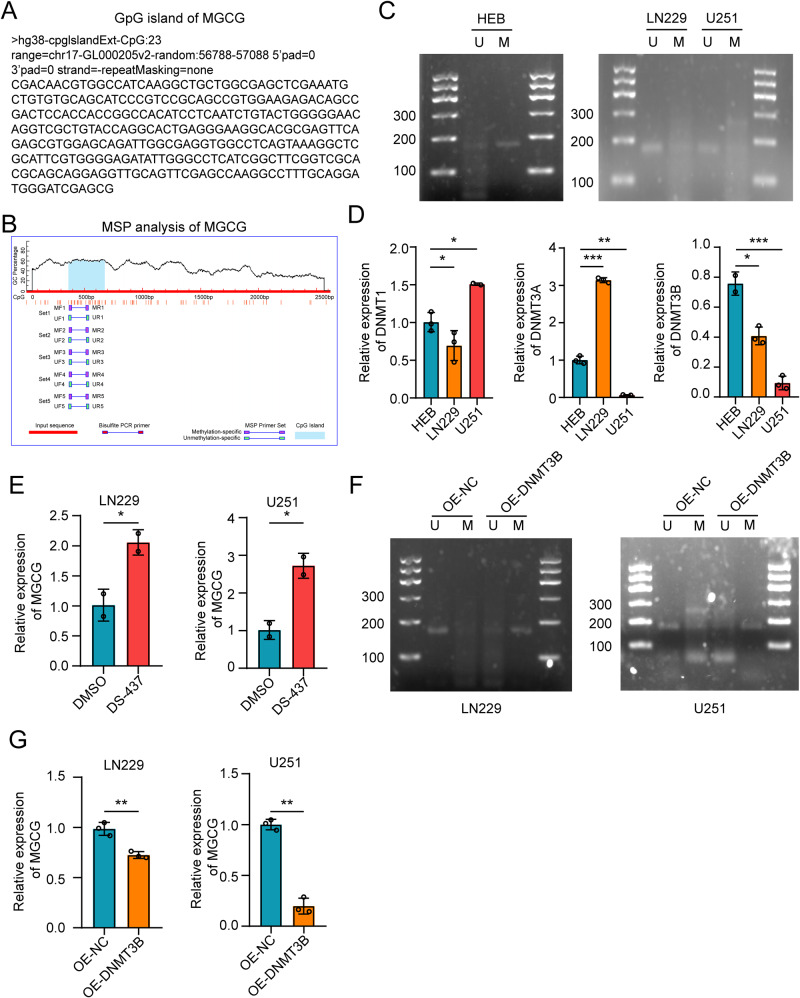
DNA methyltransferase 3B inhibits the expression of MGCG in GBM. **A** The UCSC Genome Bioinformatics Site was used to identify the CpG islands in MGCG. **B** Schematic representation of the CpG islands. The red region indicates the input sequence; the blue region indicates the CpG islands. **C** MSP analysis was performed to examine the methylation status of a CpG island in MGCG in HEB, LN229, and U251 cells. **D** The data of RT‒qPCR show the expression of DNMTs in HEB, LN229, and U251 cells. (DNMTs: DNMT1, DNMT3A, and DNMT3B). **E** RT‒qPCR assay of MGCG expression in LN229 and U251 cells after the treatment with DMSO or DS-437 for 72 h. **F** MSP analysis of CpG (MGCG) after overexpressing DNMT3B. **G** RT‒qPCR analysis of MGCG expression after overexpressing DNMT3B. Error bars, SEM from three independent experiments. *P < 0.05; **P < 0.01; ***P < 0.001 by two-tailed Student’s test.

We explored the mechanisms underlying high expression of MGCG in GBM. First, we identified the CpG islands in MGCG by using the UCSC Genome Bioinformatics Site (http://genome.ucsc.edu/) (Fig. 5A), and the data indicated a potential relationship between MGCG expression and DNA methylation. Abnormal DNA methylation has been observed in many cancers, and this disruption of epigenetic modifications may lead to aberrant gene expression in the tumor tissues [29, 30]. Using methylation analysis software (http://www.urogene.org/methprimer/), we detected a CpG island in the MGCG promoter region. Two specific primer sets were designed to detect the changes in the levels of DNA methylation of this CpG island (Fig. 5B). Next, using methylation-specific PCR (MSP), we demonstrated that GBM cells had lower methylation levels than HEB cells (Fig. 5C).

Many studies have shown that the level of DNA methylation is regulated by methyltransferases. To further explore the mechanism of the changes in methylation in MGCG, we determined the expression levels of DNA methyltransferases (DNMTs) in GBM cells. The data of RT-qPCR showed that DNMT3B expression was downregulated in GBM cells compared with that in HEB cells (Fig. 5D). We treated LN229 and U251 cells with the DNMT3B-specific inhibitor DS-437 and demonstrated that the expression of MGCG was increased in LN229 and U251 cells, indicating that DNA demethylation increased MGCG expression (Fig. 5E). Overexpression of DNMT3B led to an increase in the levels of methylation of MGCG (Fig. 5F). Moreover, overexpression of DNMT3B significantly suppressed MGCG expression in GBM cells (Fig. 5G). These findings suggested that DNMT3B promoted the expression of MGCG in GBMs.

### The MGCG/hnRNPK/ATG2A axis promotes the progression of GBM

**Fig. 6:**
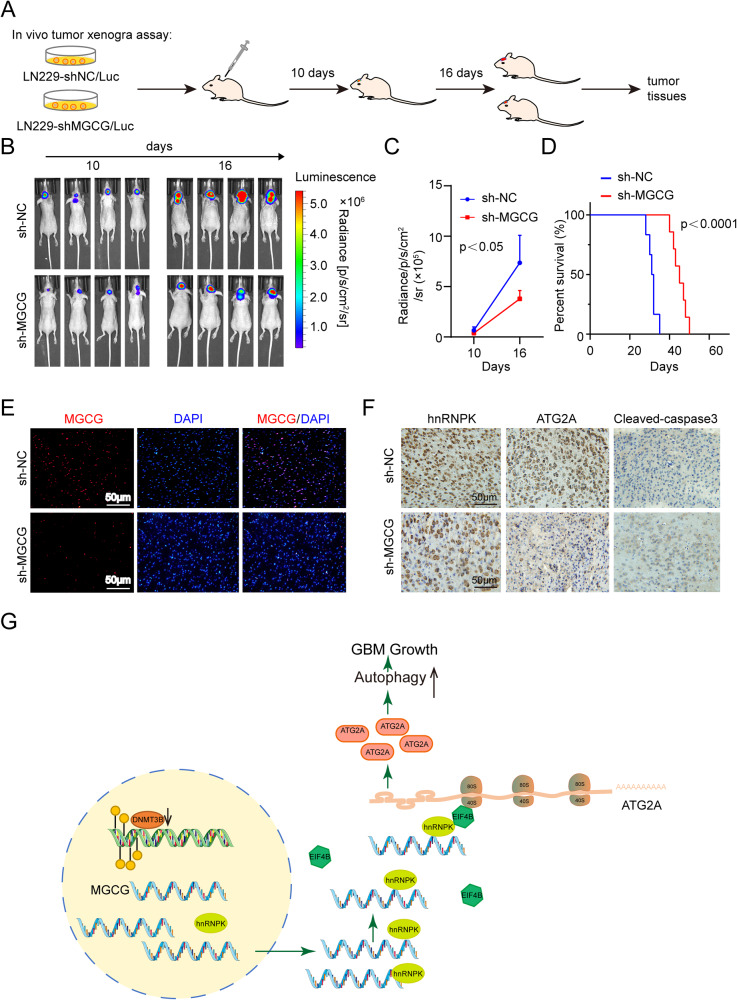
MGCG promotes GBM progression in vivo and in the clinical samples of GBM tissue. **A** Illustration of the transplantation timelines of the tumor treated with an MGCG knockdown lentivirus. Divergent arrows indicate different stages. **B** Bioluminescence images of nude mice. **C** Quantification of bioluminescent signal intensities in nude mice. **D** The Kaplan‒Meier survival curve of nude mice. **E** The expression of MGCG in the paraffin sections of a nude mouse was detected by FISH. **F** Immunohistochemical staining of hnRNPK, ATG2A, and cleaved caspase-3. **G** Hypothesis diagram illustrates the function and mechanism of MGCG in GBM.

To investigate the effect of MGCG in vivo, 2.5 × 10^5^ luciferase-labeled MGCG knockdown or sh-NC-transduced GBM cells were injected into nude mice, and the protocol of the in vivo tumor xenograft assay is shown in Fig. 6A. Tumor progression was traced by in vivo bioluminescence imaging, and xenografts carrying MGCG knockdown GBM cells displayed a significant regression in tumor growth (Fig. 6B, C). MGCG knockdown resulted in a higher survival rate of mice (Fig. 6D). The data of RNA FISH showed that MGCG expression was lower in mice injected with MGCG knockdown GBM cells (Fig. 6E). As shown by immunohistochemistry (IHC), the expression of hnRNPK and ATG2A was decreased, and the level of cleaved caspase-3 was increased after knockdown of MGCG (Fig. 6F). All these results showed that MGCG/hnRNPK/ATG2A promoted GBM progression.

## Discussion

With the development of RNA sequencing technology, transcriptome analysis has become an effective method to study the global view of cancer [31]. Glioblastoma is a highly malignant tumor with a low survival rate. Surgical resection and chemotherapy are ineffective [32]. LncRNAs are becoming important regulatory factors in various diseases, including GBM [33–38]. Recent studies have shown that lncRNA TUG1 maintains the stemness of GSCs by epigenetic inhibition of multiple neuronal differentiation-related genes [39]. The expression of HoXA11-As in the classical and mesenchymal subtypes is higher than that in the neural and proneural subtypes; thus, HOXA11-AS may be used as a biomarker to identify the molecular subtypes of GBM [40]. Therefore, it is important to elucidate the functions and mechanisms of lncRNAs in GBM.

In the present study, we demonstrated that the expression of MGCG in GBM was significantly higher than that in the normal brain tissue, identified previously unreported lncRNA MGCG, and showed that MGCG was mainly localized in the nucleus. High MGCG expression was related to poor clinical prognosis. Therefore, we investigated the malignant phenotype and mechanism of action of MGCG in GBM. The data of the functional acquisition and loss experiments showed that MGCG inhibited the development and progression of GBM in vitro and in vivo.

Using bioinformatic analysis, we found that MGCG was related to autophagy and was able to regulate the expression of the autophagy-related gene ATG2A in GBM. Autophagy plays an important role in the occurrence and development of tumors. ATG2A promotes autophagy. Autophagy-related genes play an important role in tumor progression, and some studies have shown that hypoxia-induced acetylation of PAK1 can enhance autophagy and promote the occurrence of GBM through phosphorylation of ATG5 [41]. ATG2A can promote the development of hepatocellular carcinoma through miR-541 [42]. Consistently, the present study proved that MGCG was positively correlated with the expression of ATG2A. We demonstrated that high expression of ATG2A promoted the development of tumors, was expressed at a high level in GBM, and was associated with poor prognosis.

The mechanisms of action of lncRNAs include their binding to proteins to regulate gene expression [43]; thus, we performed mass spectrometry and RIP assays and proved that MGCG was bound to hnRNPK. Many studies have shown that the members of the hnRNP family regulate gene expression through RNA-binding domains and are involved in a variety of physiological and pathological processes, such as organogenesis, erythrocyte differentiation, and carcinogenesis [44, 45]. There is growing evidence that hnRNPK is the key interaction partner of ncRNAs associated with various aspects of human health and disease [46, 47]. It has been reported that hnRNPK binds to eukaryotic translation initiation factor 4B (EIF4B) to regulate gene translation [26]. Consistently, we demonstrated that hnRNPK was bound to EIF4B to promote ATG2A translation.

LncRNA expression is affected by epigenetic regulation [48], DNA methylation is one of the modes of epigenetic regulation. The analysis of genome-wide DNA methylation patterns has proven helpful for glioma classification and diagnosis [49]. However, abnormal changes in DNA methylation patterns of lncRNAs are rarely reported. We found that the hypomethylation of MGCG promoter region caused the abnormally high expression of MGCG in GBM.

We established an orthotopic GBM tumor model and demonstrated that knockdown of MGCG inhibited the development of GBM; additionally, the expression of hnRNPK and ATG2A was elevated in the clinical samples of GBM with high expression of MGCG. The present study is the first to reveal the role of MGCG in GBM development and the details of the molecular mechanism of action of MGCG. MGCG may be a candidate for diagnosis and treatment of GBM.

The present study can be summarized as follows (Fig. 6G). We identified a new long noncoding RNA, MGCG, which acts as an oncogene and promotes GBM proliferation and migration. Next, we verified that MGCG was related to autophagy. We demonstrated that MGCG was directly bound to hnRNPK and that MGCG/hnRNPK promoted ATG2A translation. Therefore, the present study reveals a mechanism by which long noncoding RNAs regulate GBM growth and identifies potential therapeutic drug targets for GBM treatment.

## Materials and methods

### Cell lines and cell culture

Human HEB and GBM cells (LN229, U251, U87, T98G, GOS3, and TJ905) were purchased from American Type Culture Collection (ATCC). These cells were authenticated using an STR assay (Beijing Genomics institution). HEB, LN229, U251, U87, GOS3, and TJ905 cells were cultured in DMEM containing 10% FBS (Clark Bioscience, Australia); T98G cells were cultured in MEM containing NEAA and 10% FBS (Clark Bioscience, Australia) and grown in a humidified incubator at 37 °C in an atmosphere of 5% CO_2_.

### Clinical sample preparation

All fresh samples of the tumors of GBM patients (*n* = 47) and normal tissue samples (*n* = 27) (craniocerebral trauma patients) were collected from the Department of Neurosurgery of the First Affiliated Hospital of University of Science and Technology of China, and the protocol was approved by the Human Research Ethics Committee of the hospital.

### RNA extraction and quantitative real-time PCR assays

Total RNA was extracted from the normal tissue samples, GBM tissue samples, and cell lines (HEB, LN229, U251, U87, T98G, GOS3, and TJ905) using TRIzol reagent (Invitrogen, USA) according to the manufacturer’s protocol. RNA (0.5 μg) was reverse transcribed using a GoScript reverse transcription system (Promega, Beijing) and the corresponding primers. Real-time PCR analyses were performed using Trans Start Top Green qPCR SuperMix (+Dye II) (TransGen) on an ABI Q5 sequence detection system (Applied Biosystems); GAPDH was used as an internal control. All primers were synthesized by Sangon Biotech, and detailed information is shown in Supplementary Table 1.

### Nuclear-cytoplasmic fractionation

Nuclear/cytoplasmic fractionation was performed using a nuclei isolation kit (KeyGEN BioTECH) according to the manufacturer’s protocols. Nuclear and cytoplasmic RNA was extracted from LN229 and U251 cells by TRIzol reagent (Invitrogen, USA) and analyzed by real-time quantitative PCR. U6 was used as a nuclear fraction control, and GAPDH served as a cytoplasmic fraction control. All primers were synthesized by Sangon Biotech, and detailed information is shown in Supplementary Table 1.

### RNA FISH

RNA FISH analysis of the GBM cell lines and tissue samples was performed using an RNA FISH kit (RiboBio, China) according to the manufacturer’s protocols. Briefly, LN229 cells were allowed to grow until 60–70% confluency. The cells were fixed in 4% formaldehyde for 10 min, washed 3 times with PBS for 5 min, permeabilized with 0.5% Triton-X-100 in PBS for 15 min at 4 °C, and washed with PBS 3 × 5 min. The cells were incubated with a cy3-labeled MGCG probes mixture at 37 °C overnight and washed with prewarmed 2x saline-sodium citrate (SSC) 6 times for 3 min. DNA was stained with DAPI and visualized using an Olympus camera (IX73, Japan).

### Plasmids and cell transfection

The cells were transfected with MGCG siRNAs or overexpression plasmids using Lipofectamine 8000 (Beyotime Biotechnology, China) according to the manufacturer’s protocol. The MGCG knockdown and overexpression plasmids were purchased from Corues Biotechnology (Nanjing, China). GFP-LC3 adenovirus (Beyotime Biotechnology, China) was used to detected autophagosomes. Detailed information on all siRNAs and shRNAs is shown in Supplementary Table 1.

### Colony formation assay

The cells (LN229 and U251) were harvested 24 h after the transfection with si-NC, EMPTY VEC, si-MGCG, and OE-MGCG, and 500 cells were inoculated into 6-well plates that were cultured for 14 days. The resulting colonies were then washed twice with cold PBS, fixed with 4% formaldehyde for 20 min and stained for 30 min with crystal violet. The images of the colonies were then captured by an Olympus camera (BX51, Japan), and the colonies were counted by ImageJ.

### Cell migration

Cell migration was detected by a Transwell assay. After transfection with si-NC, EMPTY VEC, si-MGCG, and OE-MGCG for 24 h, LN229 and U251 cells were resuspended in 100 μL of serum-free medium. Then, the cells were seeded into the upper chamber of a Transwell assay insert (Millipore), and 600 μL of medium containing 10% FBS was added to the lower chamber. After incubation at 37 °C for 48 h, the cells on the lower side were washed three times with PBS, fixed in 4% paraformaldehyde for 20 min, and stained with crystal violet solution for 20 min. Five random fields were chosen to count stained cells for statistical evaluation using an inverted microscope (Olympus BX51, Japan), and the images were acquired.

### TUNEL assay

LN229 and U251 cells were seeded into 96-well plates. After 48 h of transfection with si-NC, EMPTY VEC, si-MGCG, and OE-MGCG, The TUNEL assays were performed using a One-Step TUNEL apoptosis kit (Beyotime Biotechnology, China) according to the manufacturer’s protocols, and the apoptosis rate was analyzed using an Olympus camera (IX73, Japan); the data were quantified using ImageJ.

### Flow cytometry

LN229 and U251 cells were seeded into six-well plates and harvested 48 h after the transfection with si-NC, EMPTY VEC, si-MGCG, and OE-MGCG. The cells were washed with cold PBS, centrifuged twice, and resuspended in Annexin V-binding buffer. Annexin V-FITC/PI staining was performed according to the manufacturer’s protocols, and the apoptosis rate was assayed by flow cytometry (FACSCanto II, America).

LN229 and U251 cells were seeded into six-well plates and culture for 48 h after the transfection with si-NC, EMPTY VEC, si-MGCG, and OE-MGCG. The cells were incubated with MDC dye for 30 min in 37 °C. The cells were washed with cold PBS, centrifuged twice, and resuspended in PBS. The fluorescence intensity was assayed by flow cytometry (FACSCanto II, America).

### Western blotting (WB)

Total protein was prepared from GBM cells using prechilled RIPA buffer with proteinase and phosphatase inhibitor cocktails (Selleck.cn, China). PVDF membranes were incubated overnight at 4 °C with primary antibodies (anti-hnRNPK, anti-ATG2A, anti-p62, anti-LC3, and anti-ACTB; (11426-1-AP, 23226-1-AP, 18420-1-AP, 14600-1-AP, 81115-1-RR) (Proteintech, China)) (1:1000) and were then incubated with an HRP-labeled secondary antibody (Sangon Biotech, China) at room temperature for 1 h. The protein bands were visualized using a chemiluminescence reagent (ECL) kit (Biosharp, China).

### Far western blot (Far WB)

The EIF4B recombinant protein (10 μg) was separated by a 10% SDS-PAGE and transferred to PVDF membrane. An equal amount of BSA was also loaded as a negative control. The proteins on the membrane were denatured and renatured in AC buffer (100 mM NaCl, 20 mM Tris-HCl pH 7.6, 0.5 mM EDTA, 10% glycerol, 0.1% Tween-20, 2% non-fat milk and 1 mM DTT) by gradually reducing the guanidine-HCl concentration. After blocking the membrane with 5% non-fat milk in 1 × TBST buffer at RT for 1 h, the membrane was incubated with 1 μg/ml recombinant hnRNPK protein at 4 °C overnight. After that, the membrane was incubated with anti-hnRNPK antibody (Proteintech, China) (1:1000) in the TBST buffer containing 3% milk at RT for 1 h. Finally, the HRP conjugated goat anti-rabbit secondary antibody (Sangon Biotech, China) (1:4000) was incubated for another 1 h and chemiluminescent detection was performed.

### Immunofluorescence (IF)

The cells were plated in a glass bottom cell culture dish (Biosharp, China). The cells were stained using standard procedures. A primary antibody against hnRNPK was diluted in 1% BSA in PBS. After overnight incubation at 4 °C, the cells were washed three times with PBS and incubated with FITC-labeled anti-IgG antibodies (Sangon Biotech, China) for 1 h at room temperature. DNA was stained with DAPI (Sigma, USA) and visualized using a laser scanning confocal microscope (LSCM) (ZEISS LSM800, Germany).

### RNA pulldown

RNA pulldown was performed using 5′-biotinylated MGCG oligos and a negative probe according to a previously described method. Briefly, GBM cells were cross-linked in a UV cross-linker (UVP) at a power of 200 mJ. The cells were granulated and resuspended in RIPA buffer (50 mM Tris-Cl, pH 8.0, 150 mM NaCl, 5 mM EDTA, 1% NP-40, 0.1% SDS, 1 mM DTT, 1× protease inhibitor cocktail (Roche, Switzerland), and 0.1 U/μL RNase inhibitor) for 10 min on ice, harvested, and sonicated for 20 min. The cell lysate was centrifuged at 14,000 × *g* for 20 min. Subsequently, biotinylated MGCG oligonucleotides (100 pmol) or negative oligos (as a control) were added to the supernatant at 4 °C for 2 h. Subsequently, 50 μL blocked M-280 streptavidin Dynabeads (Thermo, USA) were added to 100 pmol of biotin-DNA oligonucleotides. Then, the mixture was rotated for 4 h at 4 °C. The beads were captured using magnets (Life Technologies) and washed three times with RIPA buffer supplemented with 500 mM NaCl. RNA and protein were extracted from the beads and used for further analysis.

### RNA immunoprecipitation assay (RIP)

Briefly, 10^7^ cells were washed with cold 1× PBS three times and harvested in ice-cold lysis buffer (10 mM HEPES, pH 7.4, 200 mM NaCl, 30 mM EDTA, 0.5% Triton-X-100, 100 units/mL RNasin Plus RNase inhibitor, 1.5 mM DTT, 1× protease inhibitor cocktail (Roche, Switzerland)) and sonicated for 15 min with an ultrasonic disruptor. The cell suspension was centrifuged at 14,000 × *g* for 20 min at 4 °C, and the supernatant was collected. A 200-μL sample of the supernatant was saved as an input. Subsequently, an anti-hnRNPK (1:1000) (Proteintech, China) or IgG (1:1000) (Sangon Biotech, China) antibody was added to the cell suspension and incubated for 2 h at 4 °C. Blocked Protein G Dynabeads (Thermo, USA) were washed three times in RIPA buffer and added to the cell suspension for binding for at least 4 h at 4 °C. The antibody–protein G bead complexes were washed five times with lysis buffer and digested with 30 μg of proteinase K at 55 °C for 30 min. Finally, immunoprecipitated RNA was purified and detected by quantitative real-time PCR.

### Immunohistochemistry (IHC)

In brief, formalin-fixed, paraffin-embedded tissue sections were incubated at 65 °C for 60 min, dewaxed in xylene, rinsed in graded ethanol, and rehydrated in double-distilled water. For antigen retrieval, the slides were pretreated by steaming in sodium citrate buffer for 8 min at 95 °C. After washing with PBS for 3 min, the sections were immunostained with primary antibodies against hnRNPK, ATG2A, and cleaved caspase-3 and incubated at 4 °C overnight. After being washed in PBS, the tissue samples were overlayed with an anti-mouse/rabbit HRP-labeled polymer for 60 min. The staining reactions were performed by overlaying the tissue samples with DAB chromogen solution, and the samples were incubated for ~1 min to allow for proper brown color development.

### Methylation-specific PCR

Genomic DNA was extracted from HEB, LN229, and U251 cells with a QIAamp DNA Mini kit (Qiagen, Germany). Purified DNA was exposed to bisulfite using an EpiTect bisulfite kit (Qiagen, Germany) according to the manufacturer’s protocol. Methylation-specific PCR (MSP) of bisulfite-transformed DNA was performed according to a nested two-stage PCR method. Amplified PCR products were separated by 3% agarose gel electrophoresis and visualized with Gel Red. All primers were synthesized by Sangon Biotech, and detailed information is shown in Supplementary Table 1.

### Tumor xenograft model

Female BALB/c nude mice (aged 4–5 weeks, 18–20 g) were purchased from GemPharmatech Jang Su and reared in laminar airflow cabinets under specific pathogen-free conditions. Subsequently, 1 × 10^7^ LN229 cells were stably transfected with sh-MGCG or sh-NC, and a total of 3 × 10^5^ LN229 cells per mouse were stereotactically injected into the brain (NC *n* = 4, sh-MGCG (*n* = 4)). Intracranial tumors were assessed by bioluminescence imaging. Mice were sacrificed, and the brain tissue was isolated. The samples of the mouse brain tissue were embedded in paraffin and sectioned at a thickness of 4 μm for immunohistochemistry assays. All animal studies were approved by the Institutional Animal Care and Use Committee of the First Affiliated Hospital of USTC.

### Statistical analysis

All data were analyzed with GraphPad Prism software (version 9.0) and expressed as the mean ± SD from three independent experiments. All experiments in this work were replicated at least three times. The differences between two groups were analyzed by Student’s t-test, and one-way analysis of variance (ANOVA) was used for multiple groups. The Kaplan–Meier method was used to assess the overall survival rate of patients. Differences with *P* < 0.05 were considered statistically significant.

## Supplementary information

Original Data File

aj-checklist

Supplementary information

Table S1

## Data Availability

The data generated or analyzed during this study are included in this article and its additional information files.
