# Supplementary material for: MGCG regulates glioblastoma tumorigenicity via hnRNPK/ATG2A and promotes autophagy
Source: Cell Death Dis. 2023 Jul 17;14(7):443. doi: 10.1038/s41419-023-05959-x (PMC10352271; doi:10.1038/s41419-023-05959-x)
Supplement: Supplementary file 1 — Original Data File [file 41419_2023_5959_MOESM1_ESM.docx]

**original data files**

ACTB


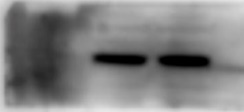

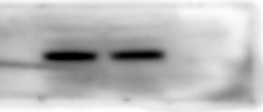


ATG2A


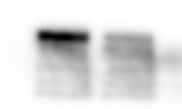

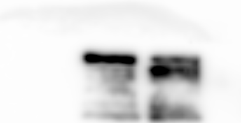


**Fig. 3H, J**


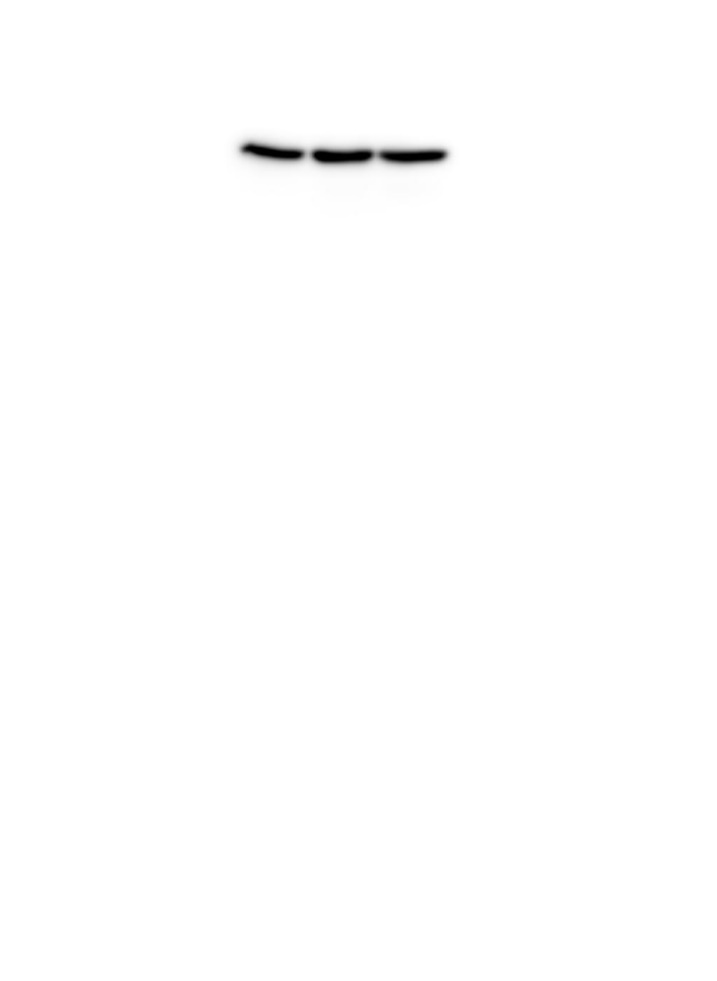


ACTB


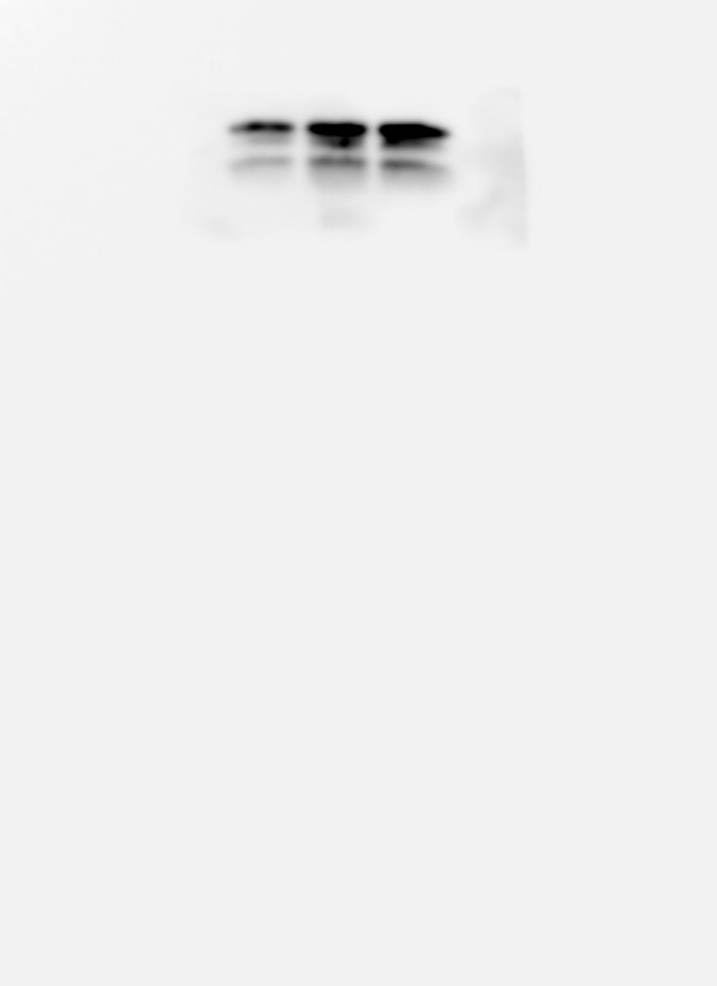


LC3I

LC3II


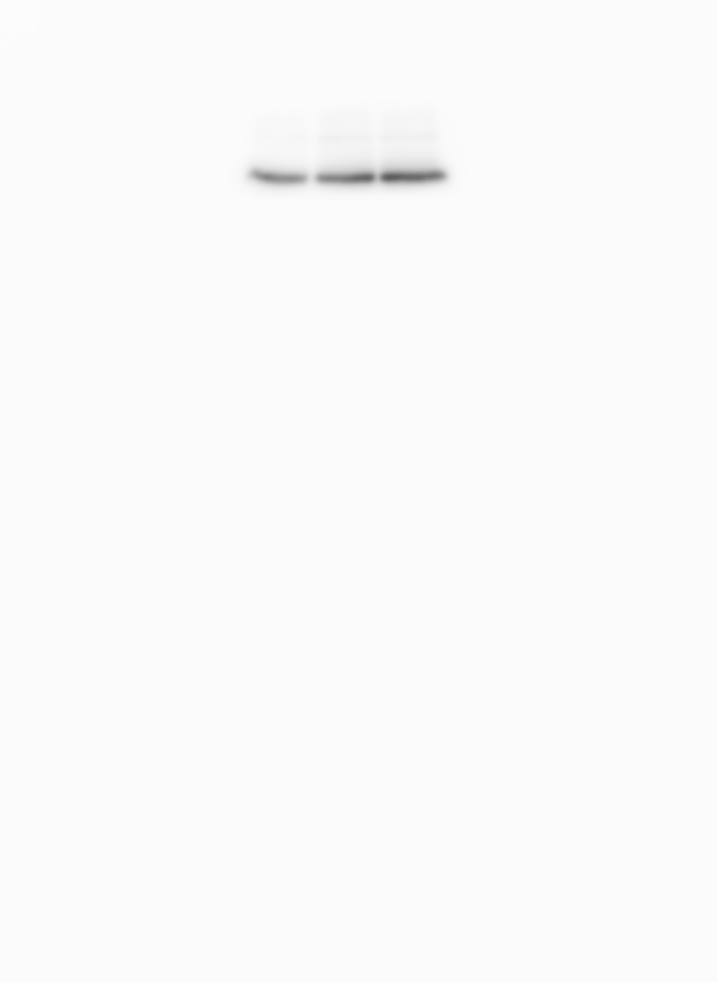


P62

**Fig. 2J**


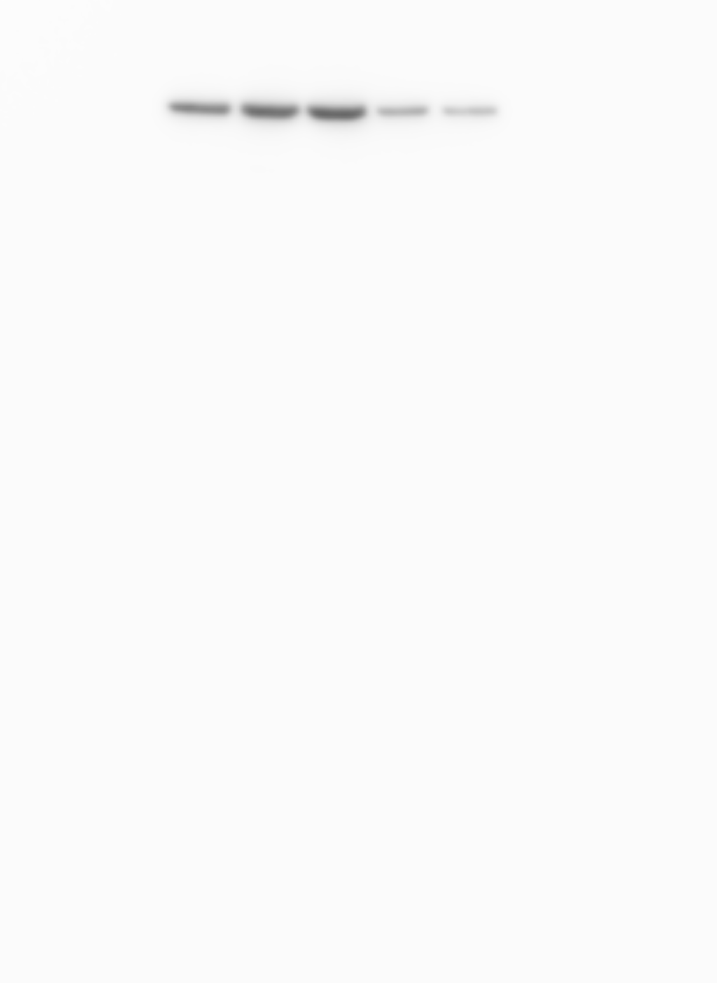

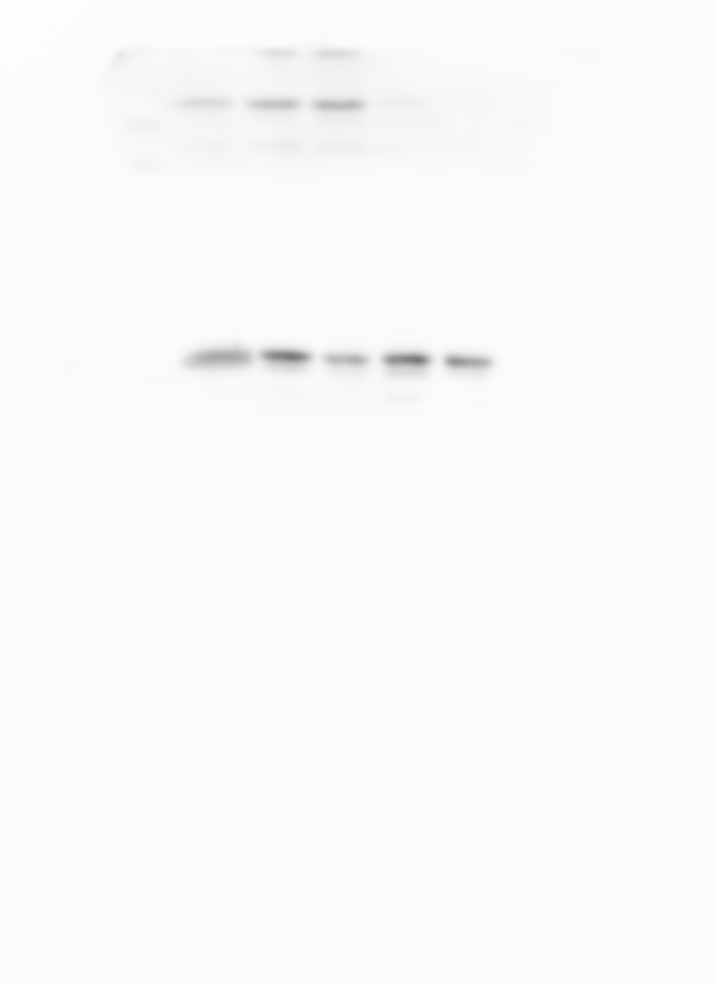


P62

LC3I

LC3II

ACTB


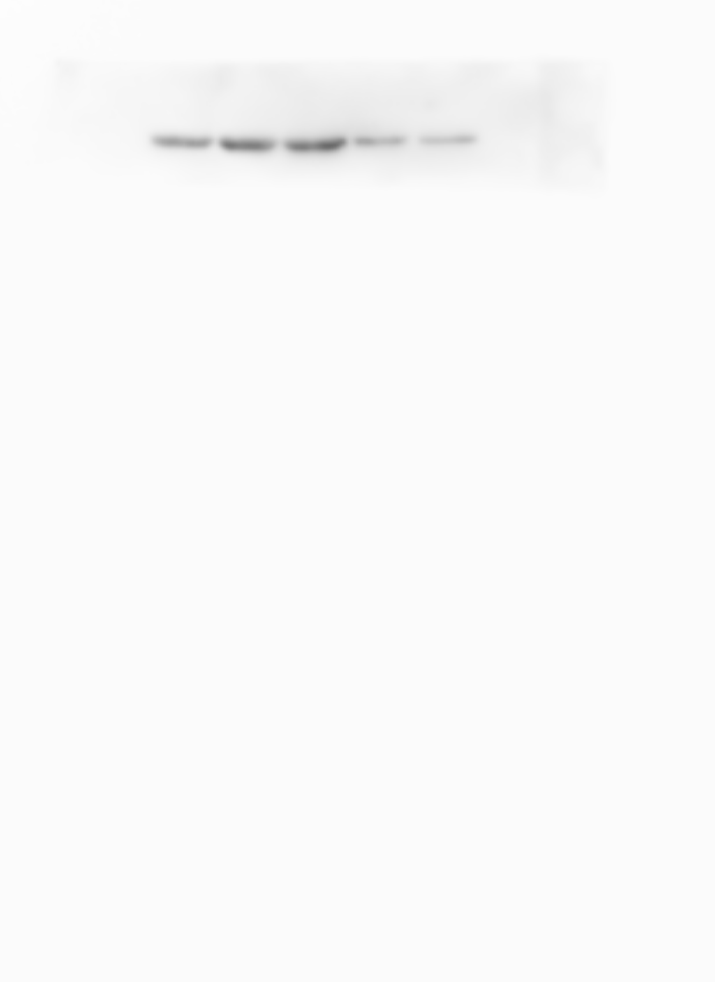


**Fig. 3D
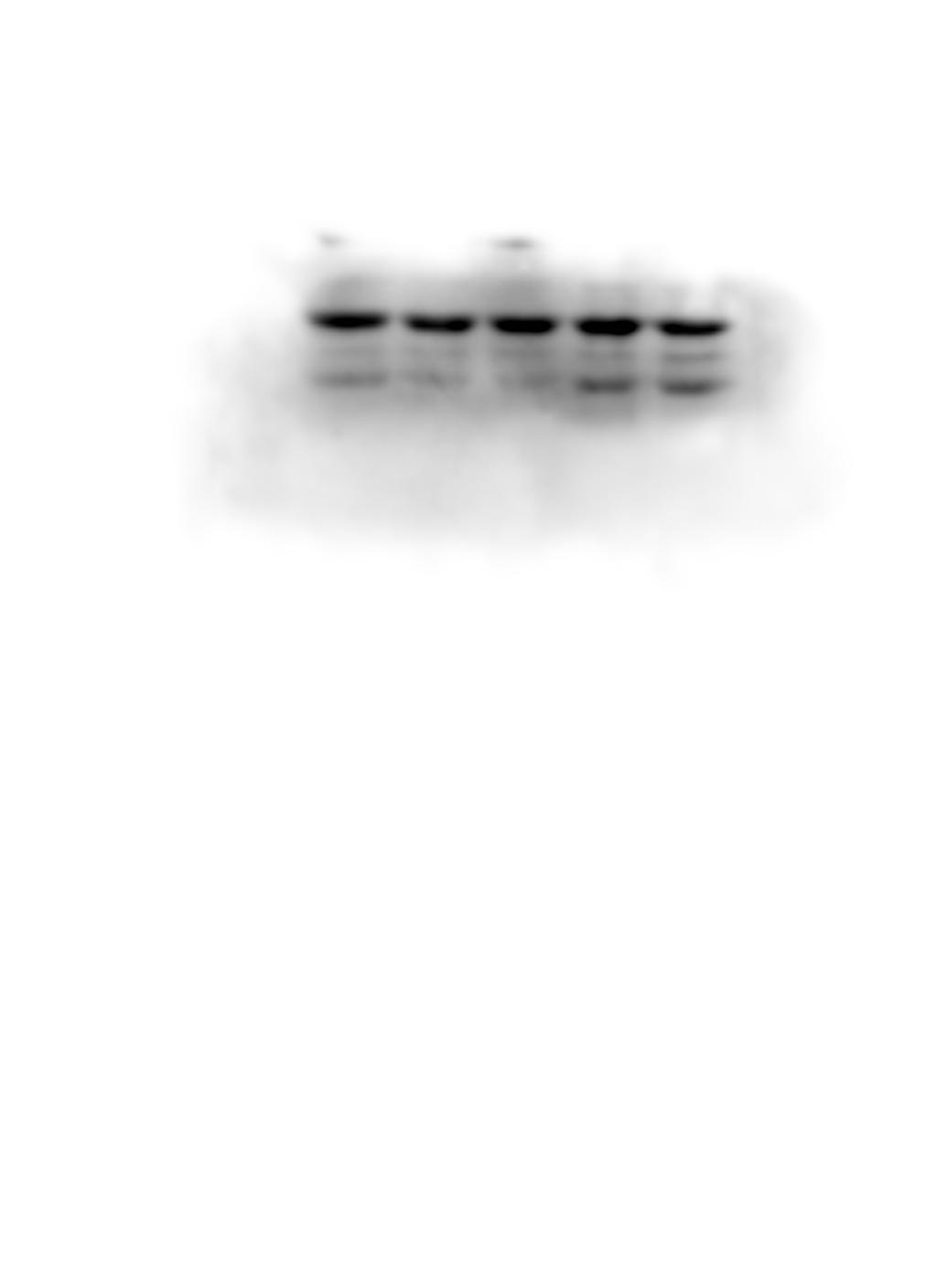
**

ACTB


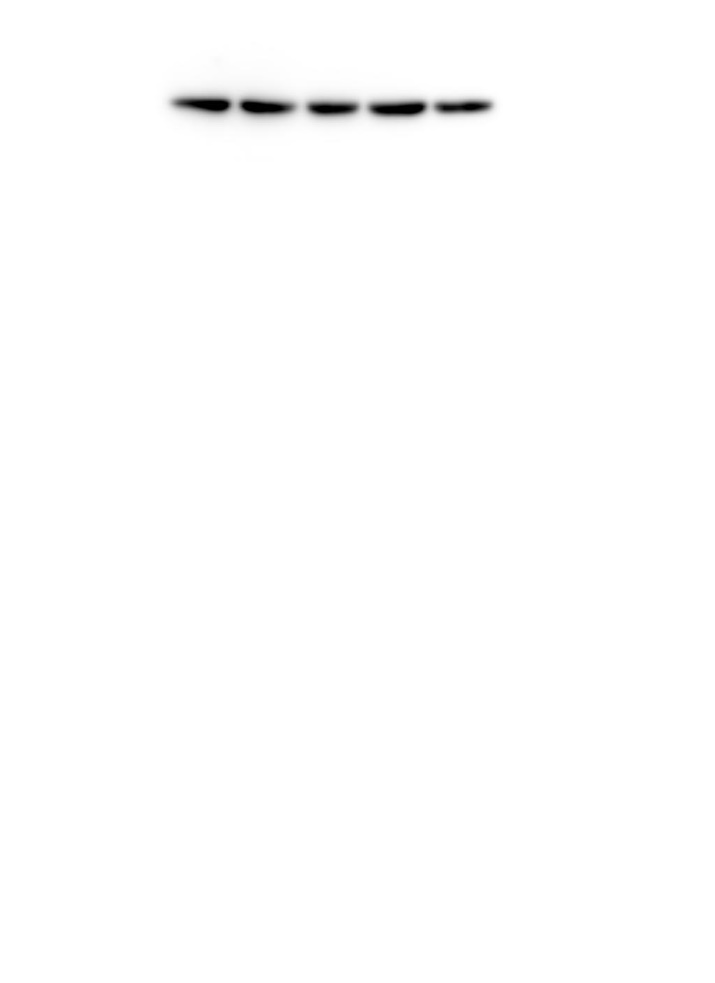

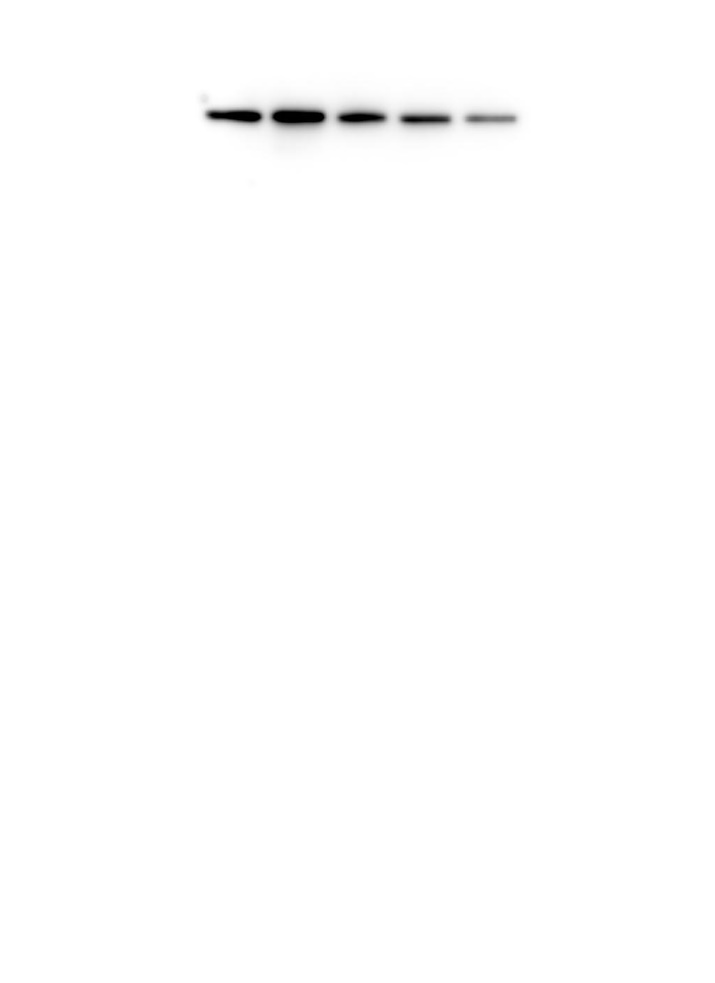


ATG2A

**Fig. 3B**

ACTB

P62


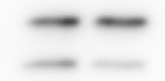


LC3I

LC3II


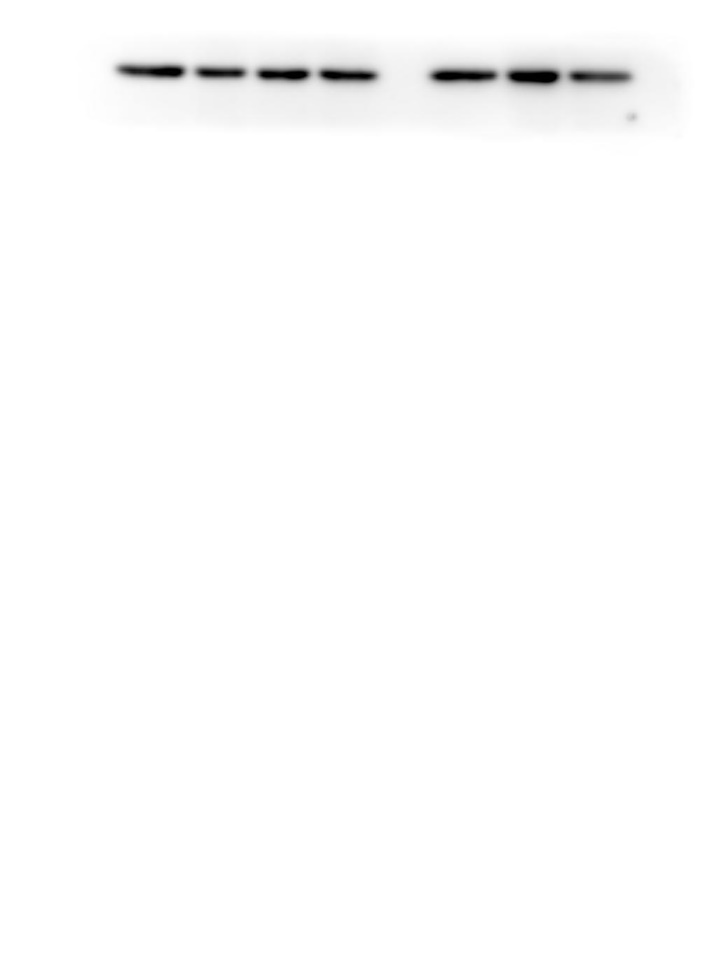

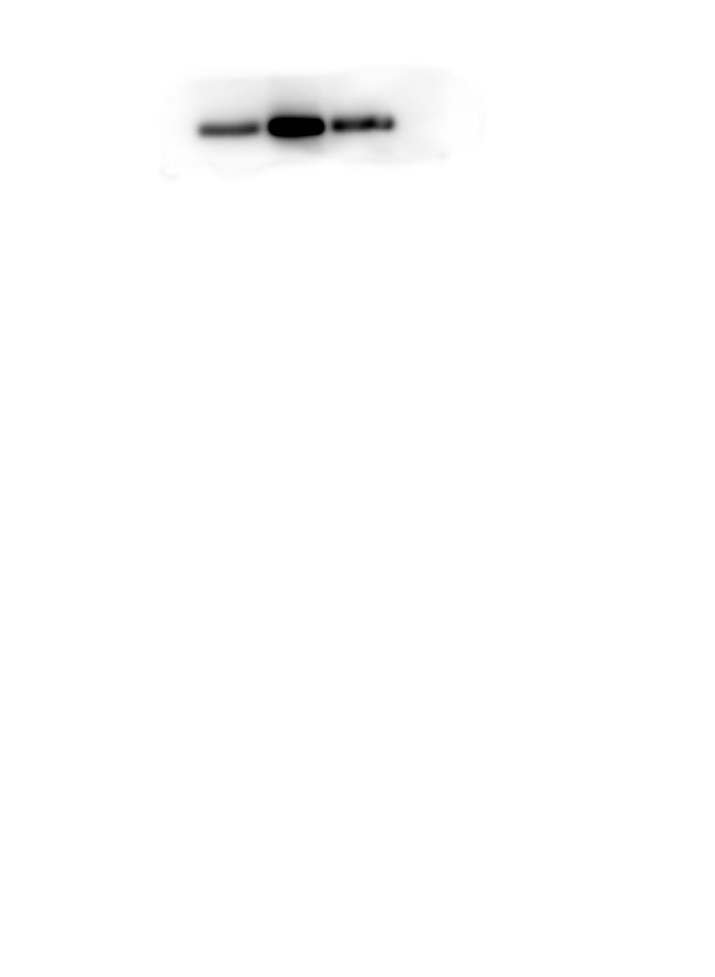

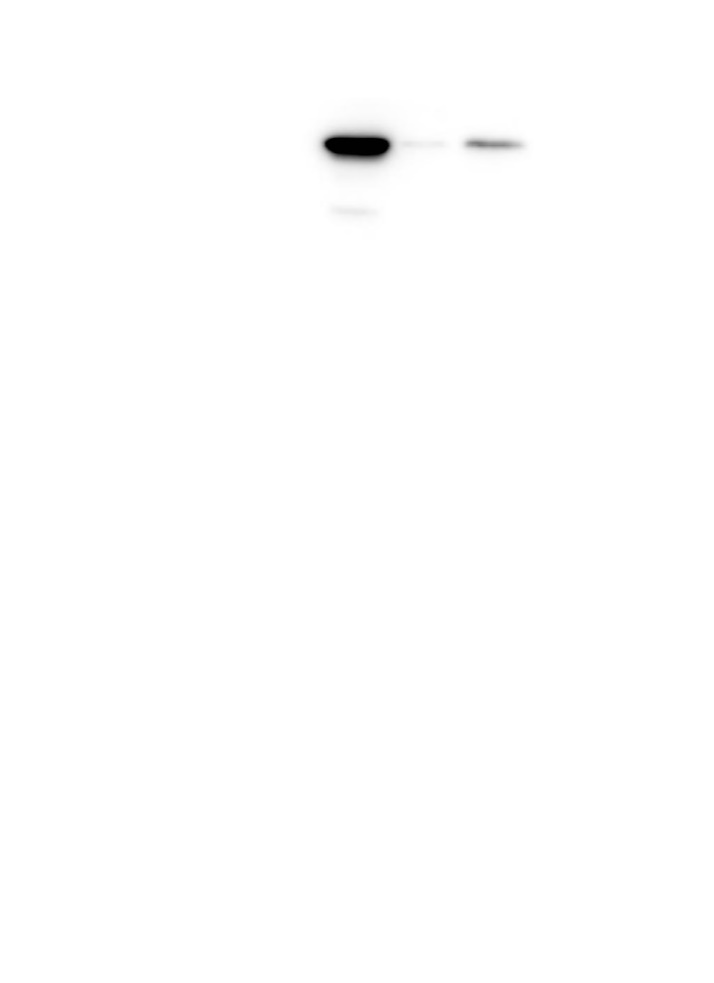


**Fig. 4J**

hnRNPK

hnRNPK

**Fig. 4I**


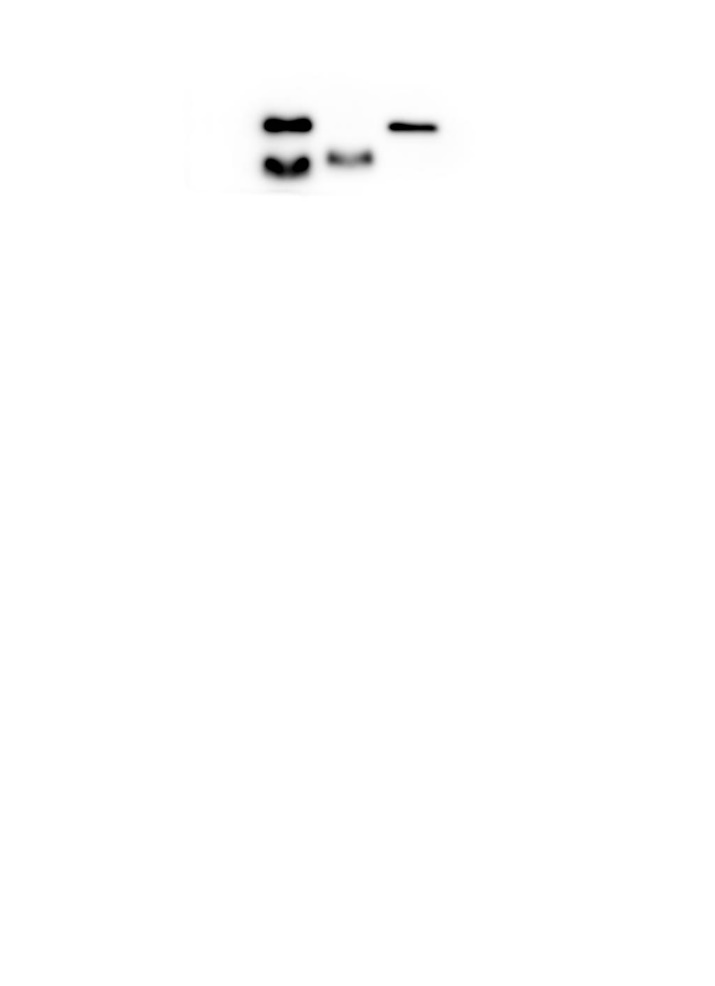


**Fig. 4G**

ATG2A

ACTB


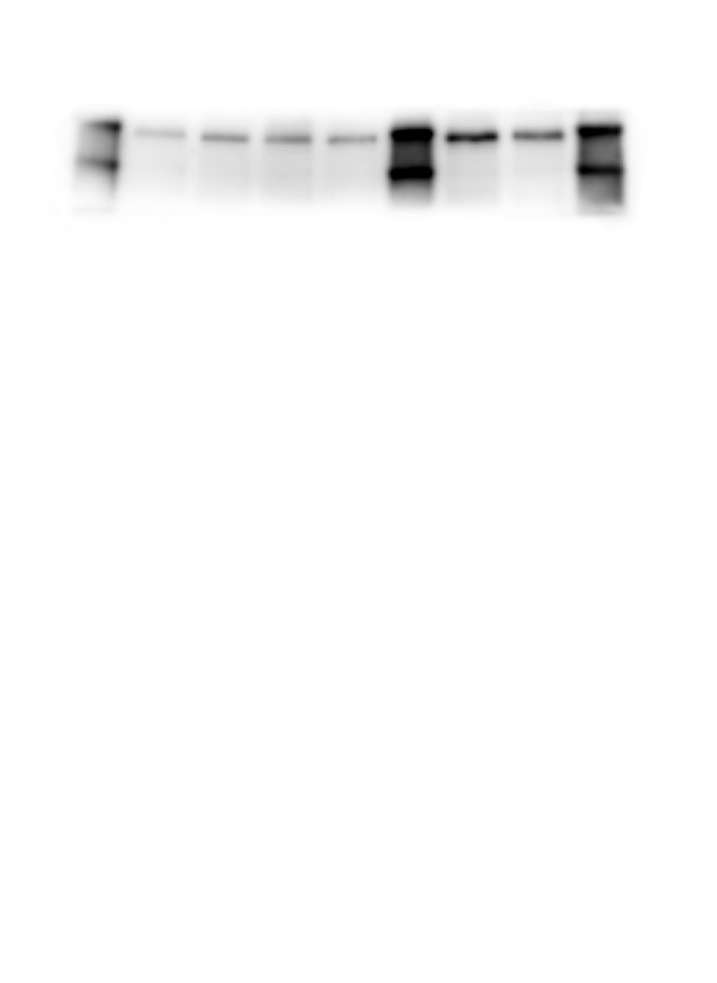

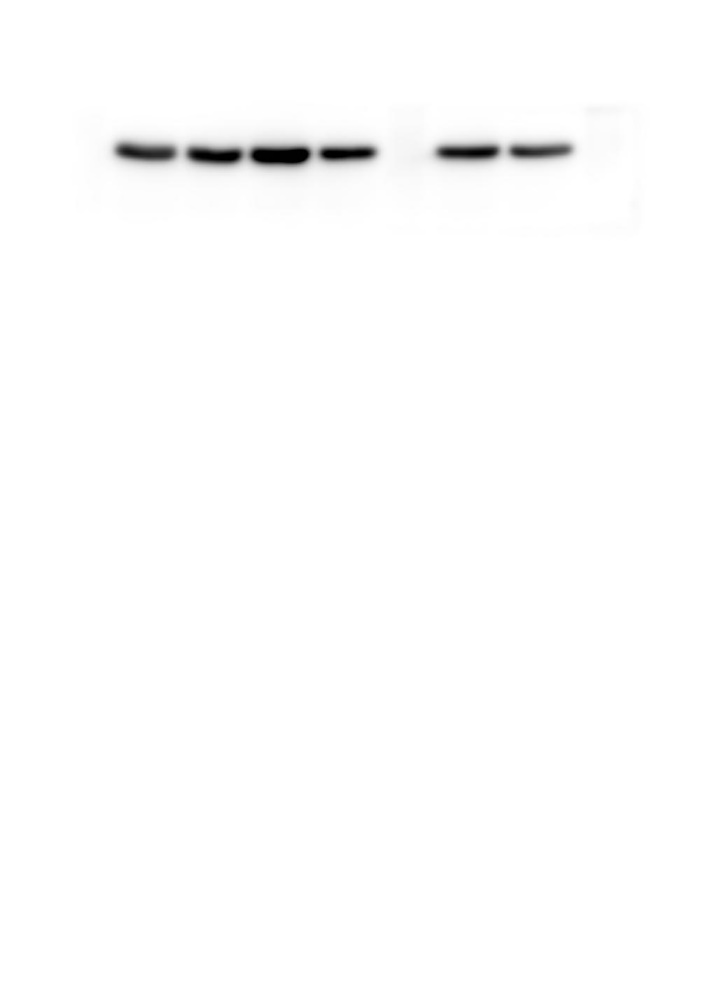


**Fig. 4E**


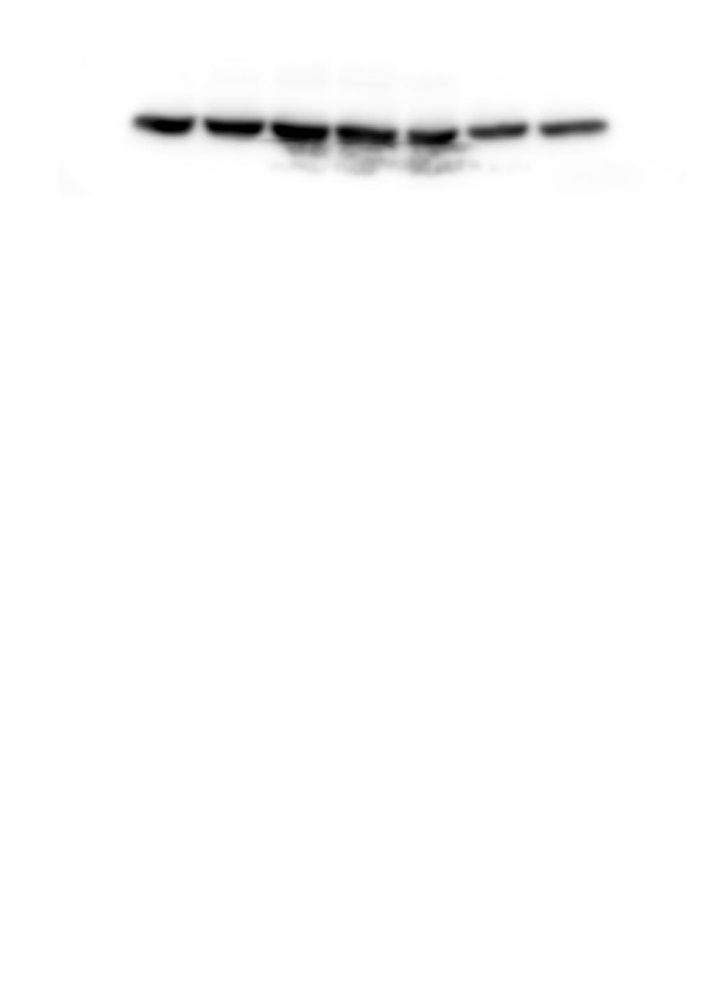


ACTBv

hnRNPK


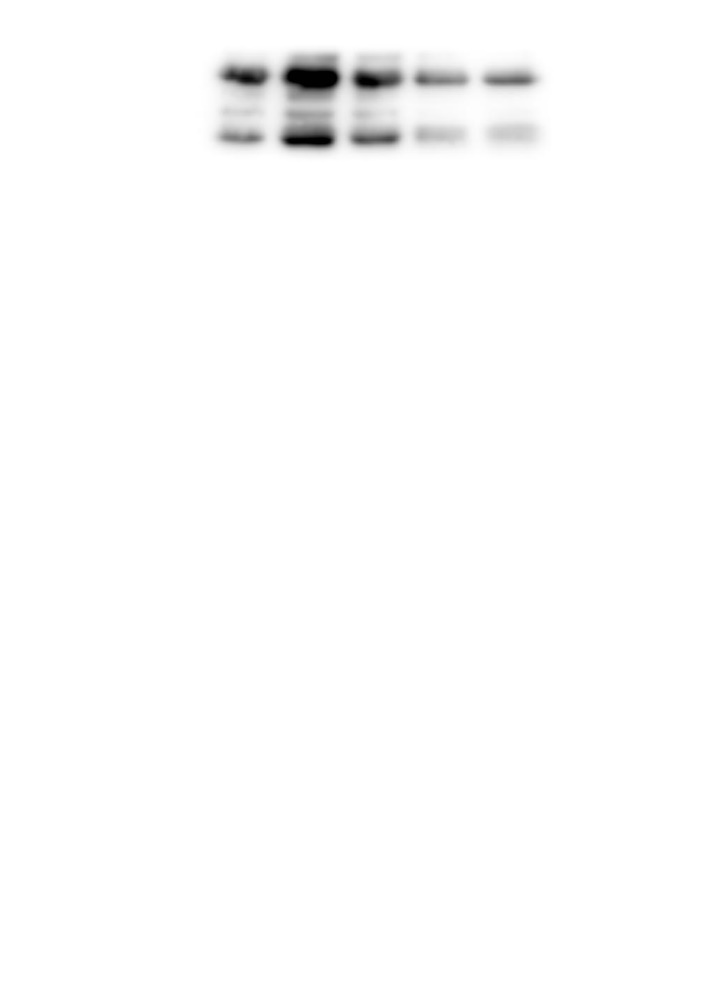

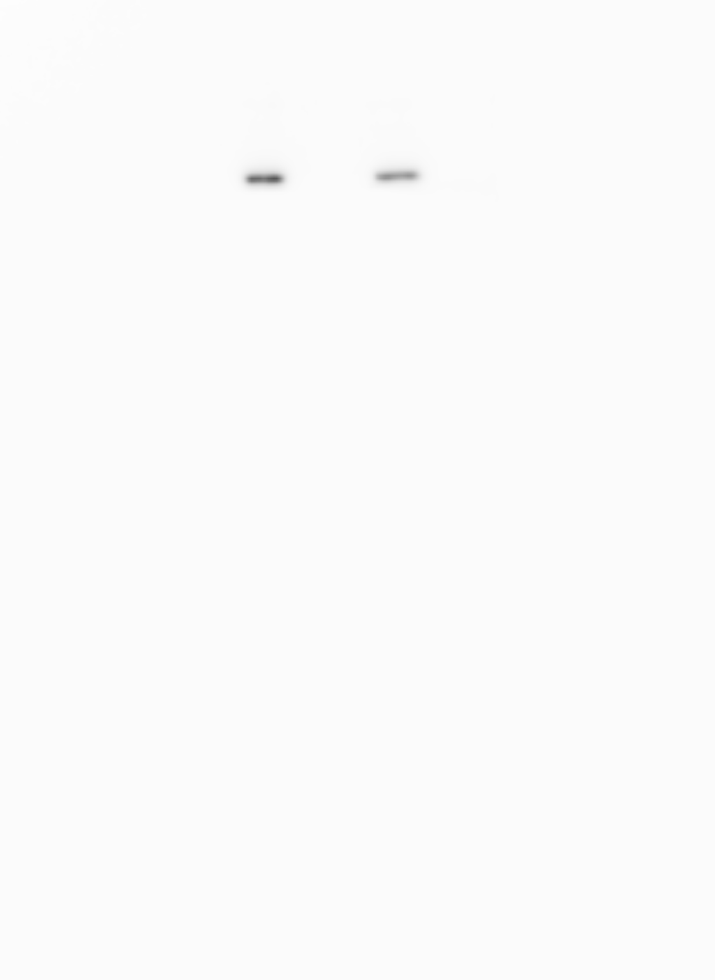


**Fig. 4C**

hnRNPK

**Fig. 3N**


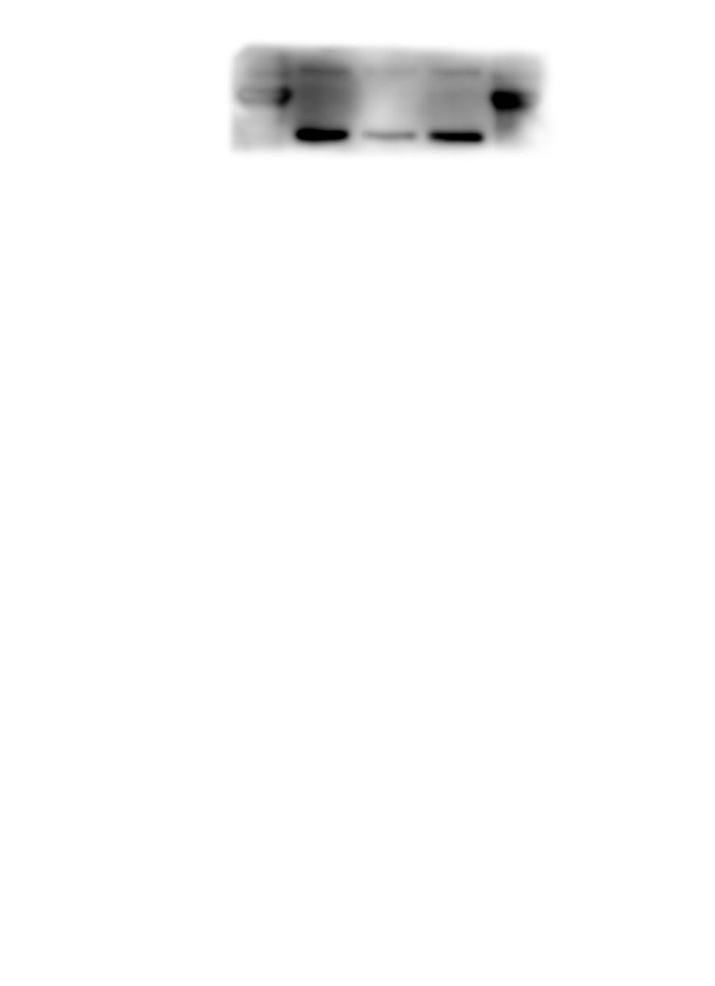


P62


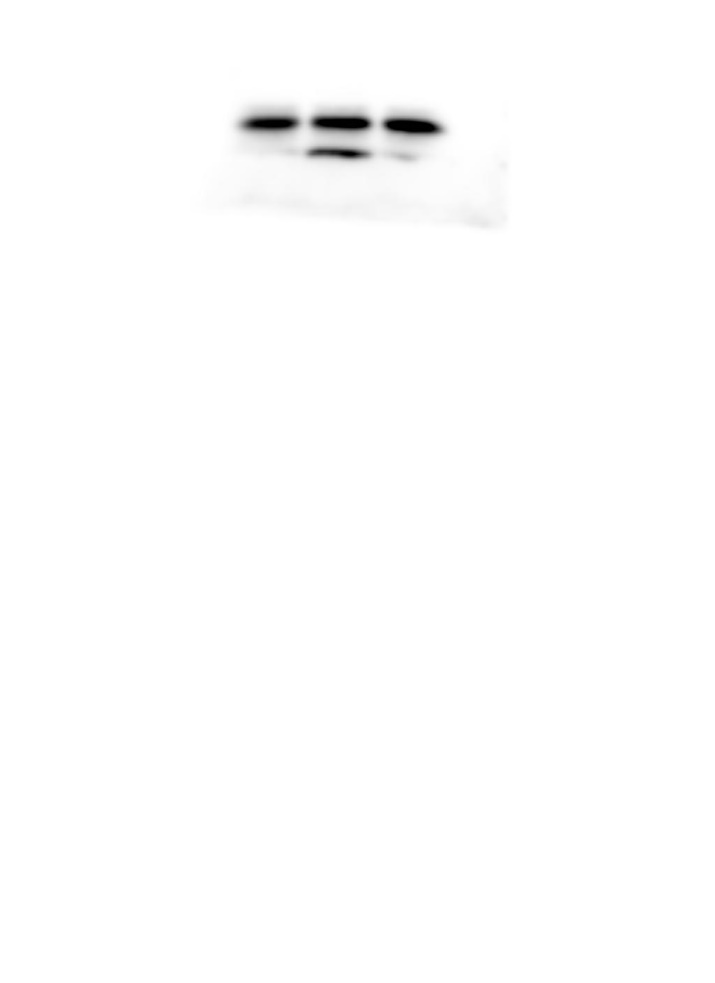


LC3I

LC3II

ACTB


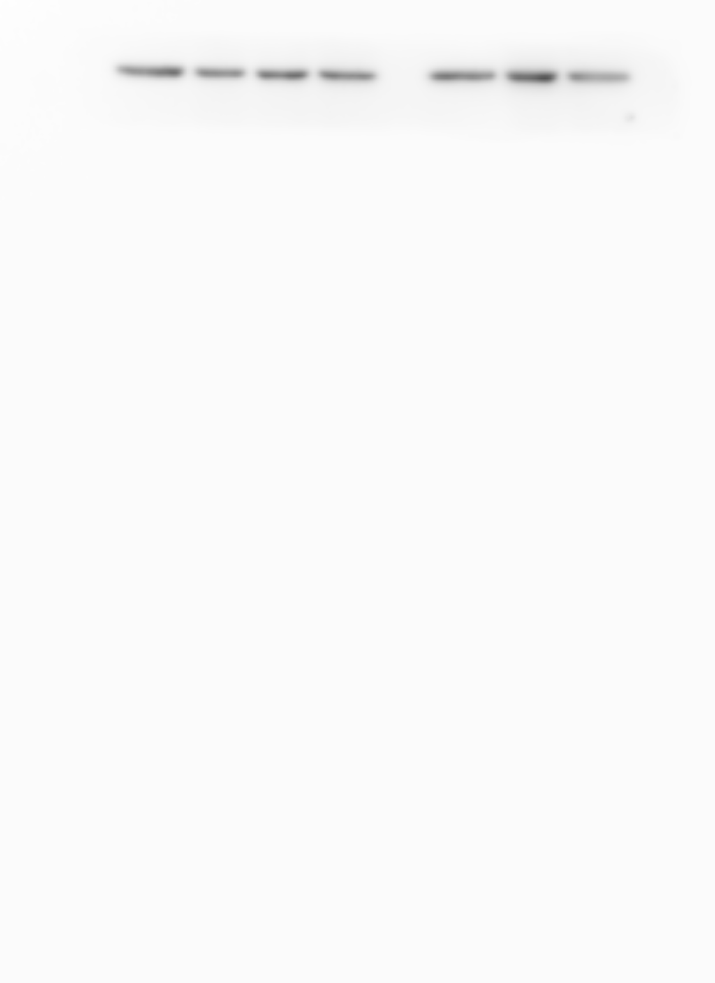

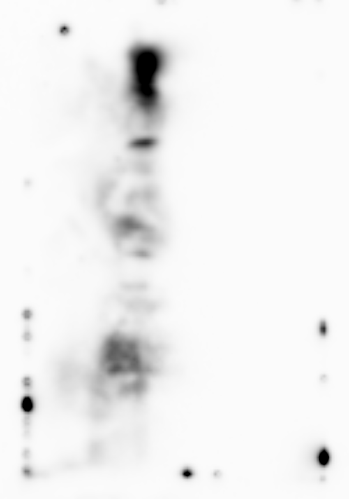


EIF4B


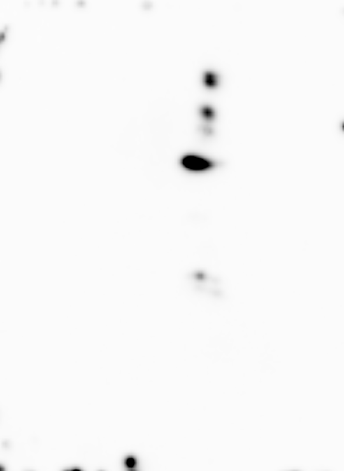


**Fig. 4K**

hnRNPK


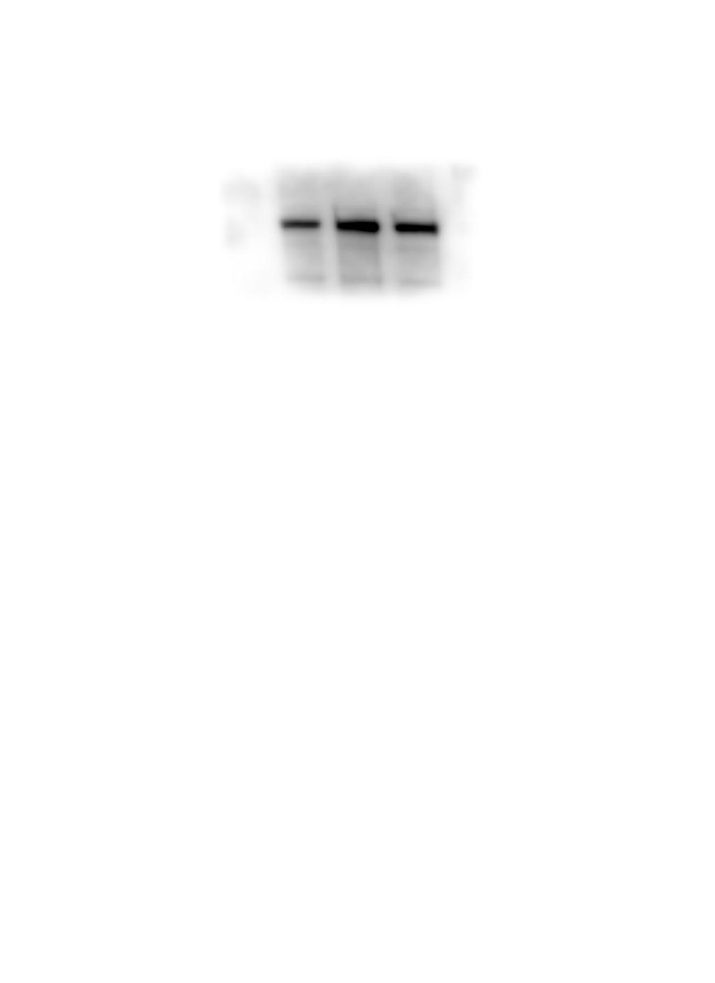


**Fig. 4N**

ATG2A

ACTBv

hnRNPK


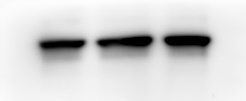

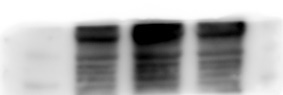

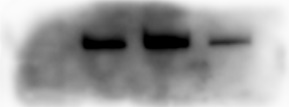

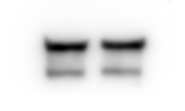

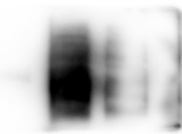


**Fig. 4L**

ACTBv

ATG2A

**Fig. S3G
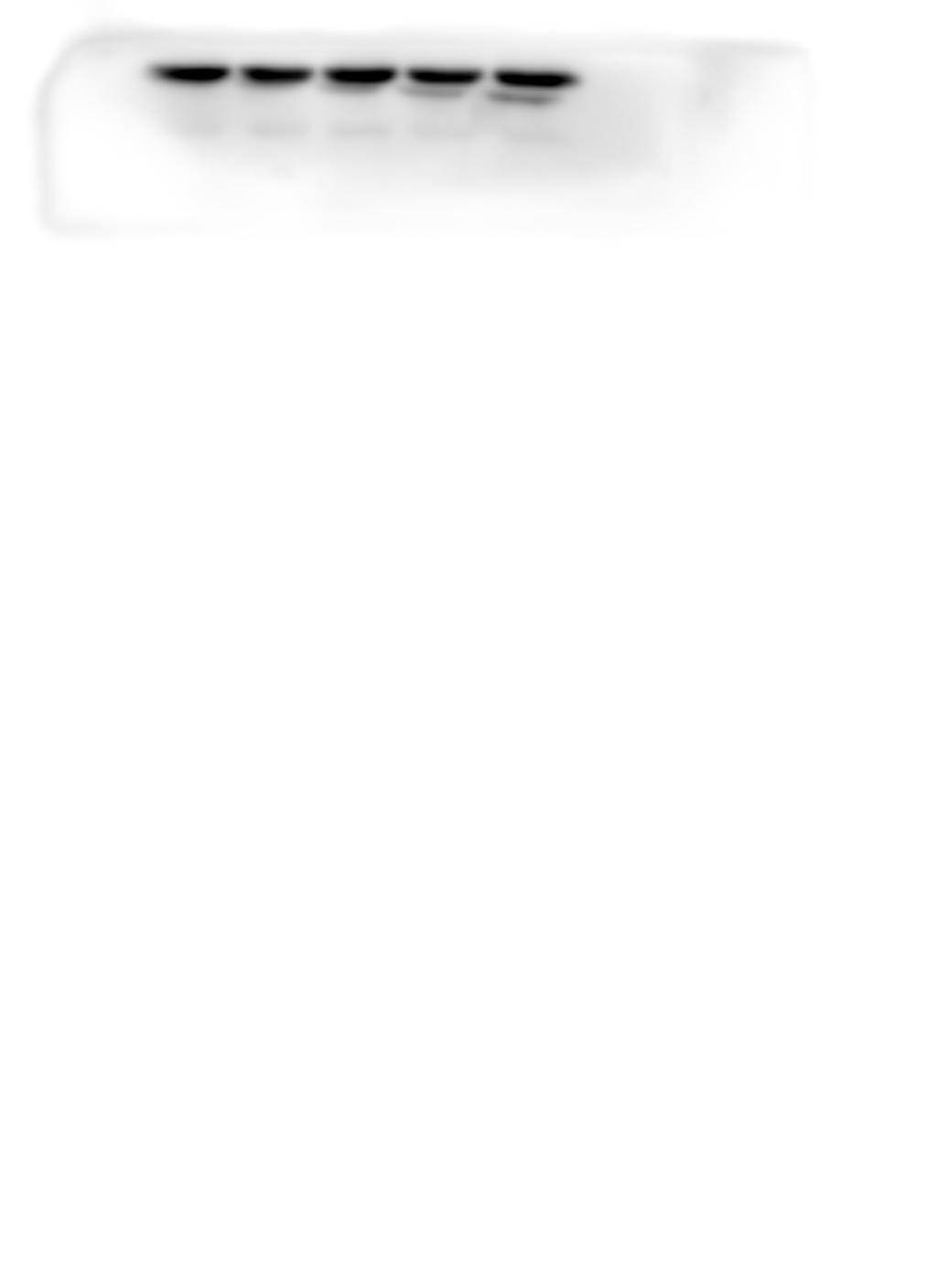
**


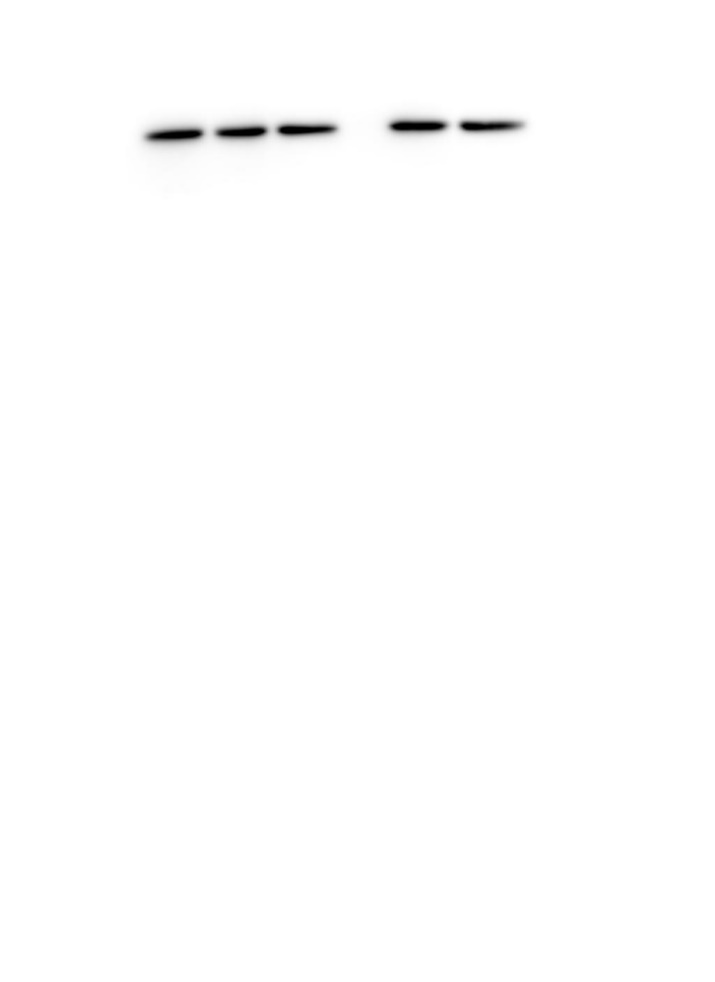


ACTB


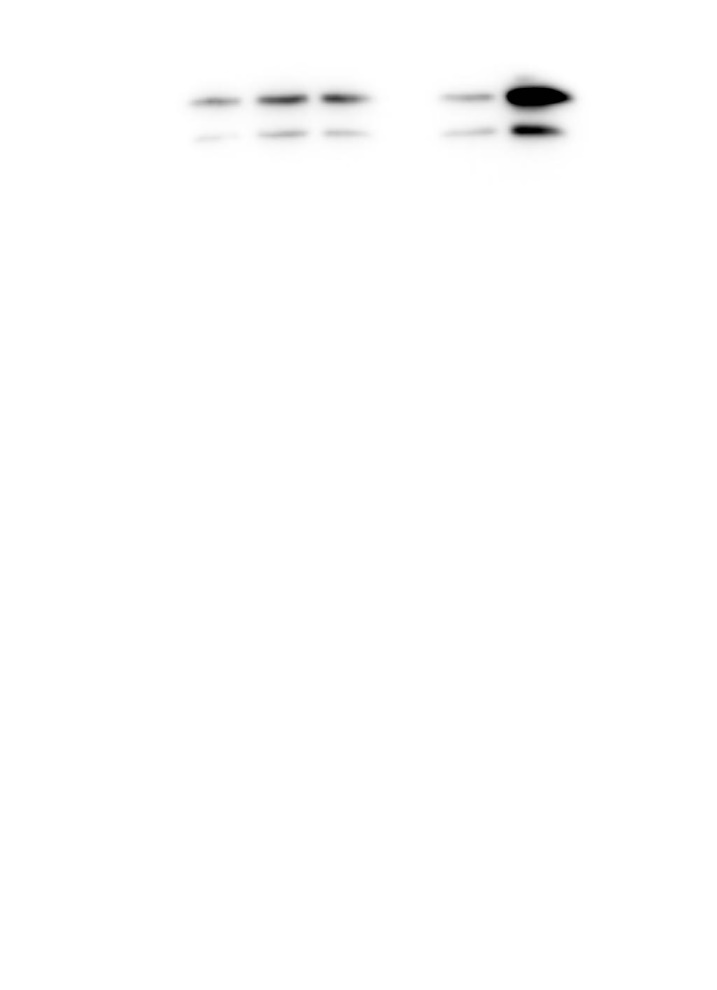


LC3I

LC3II


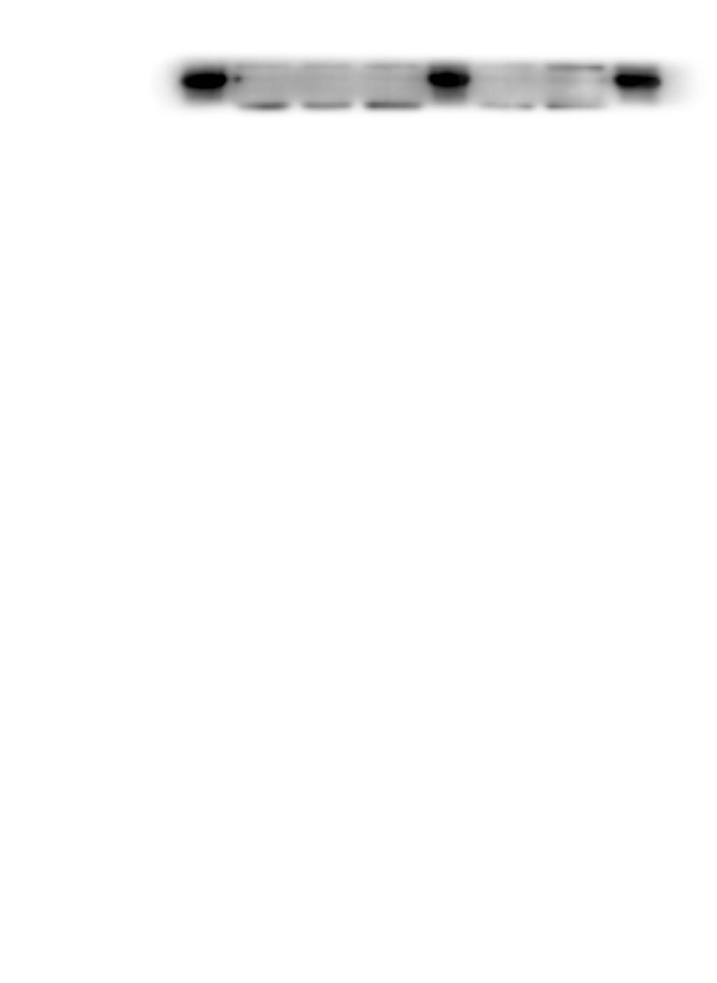


P62


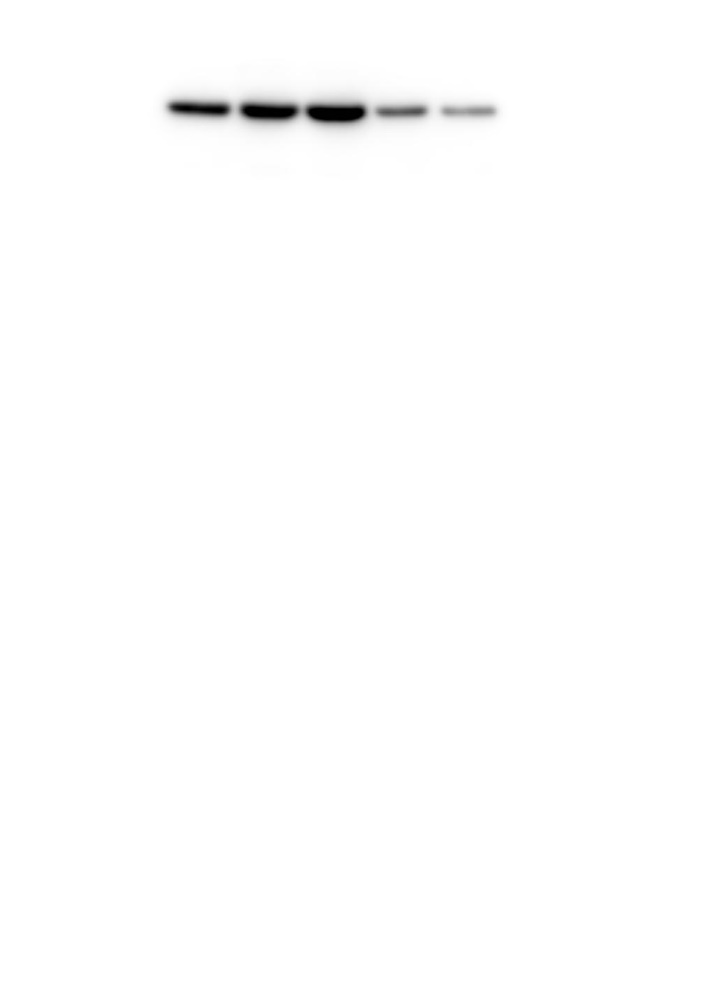

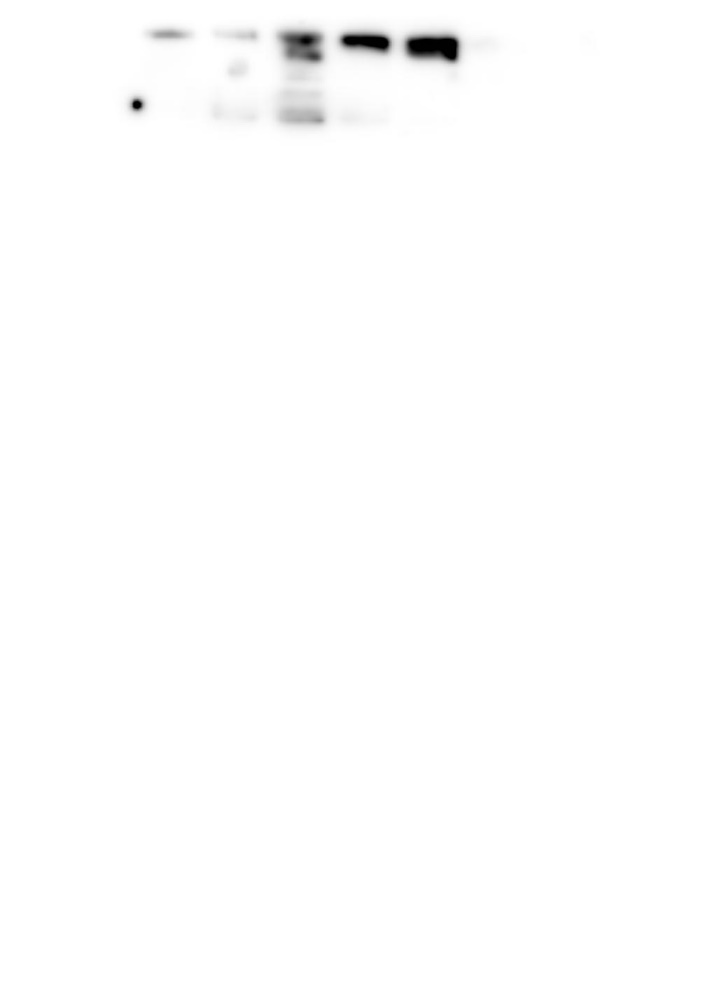


**Fig. S3C
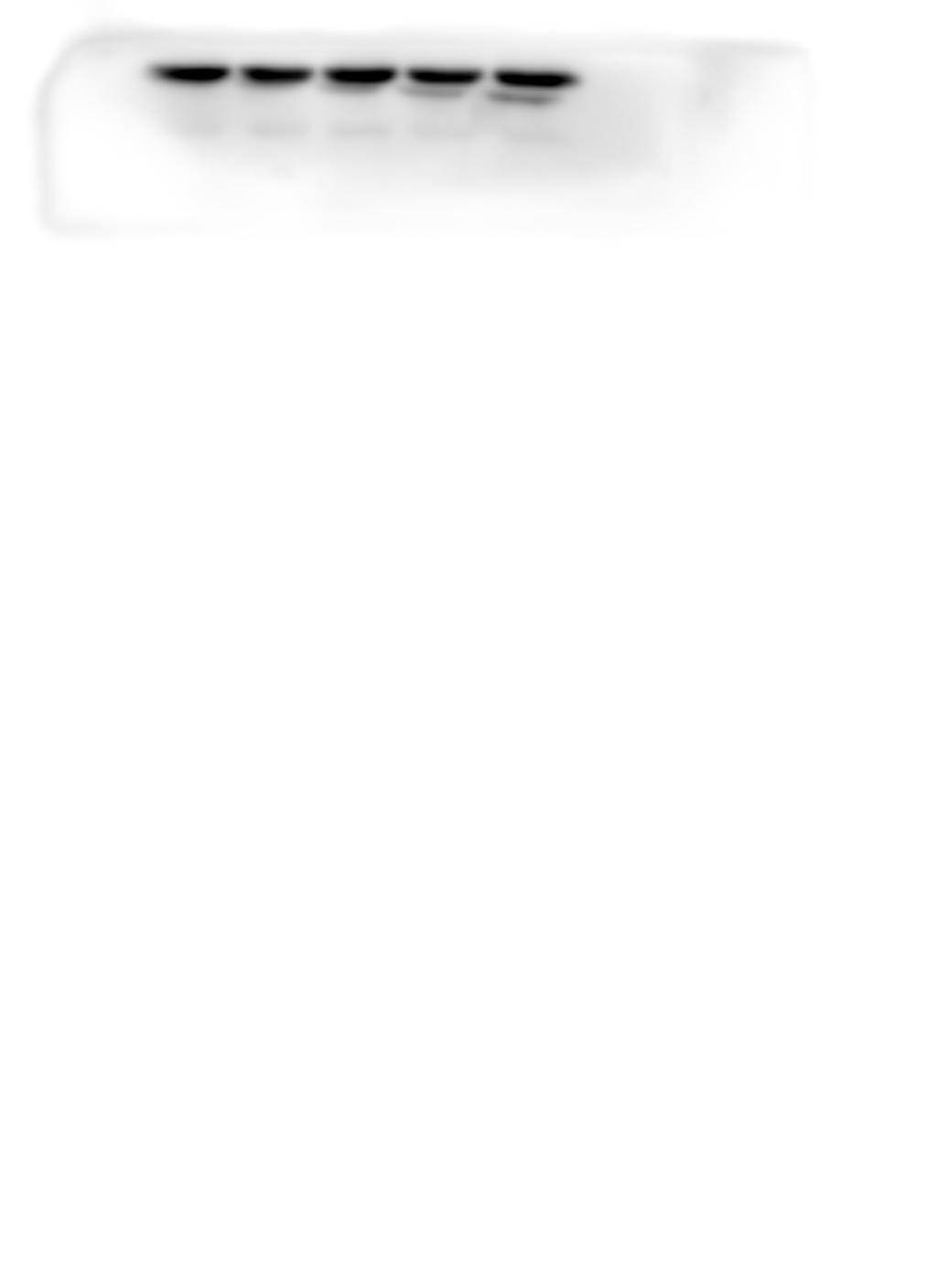
**

ACTB

ATG2A


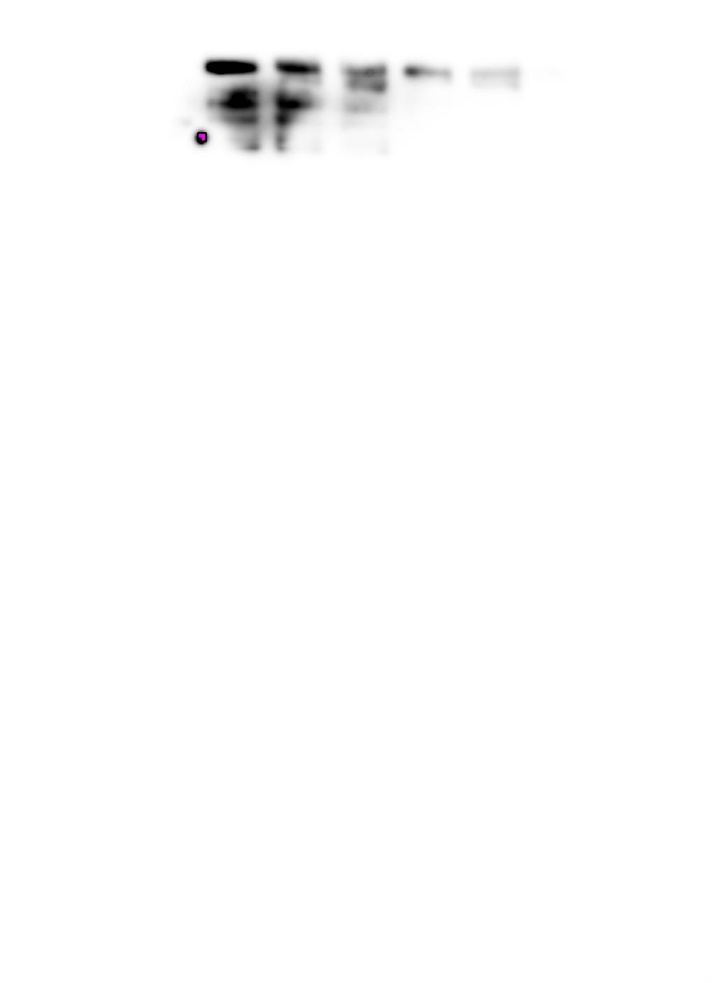


ATG2A


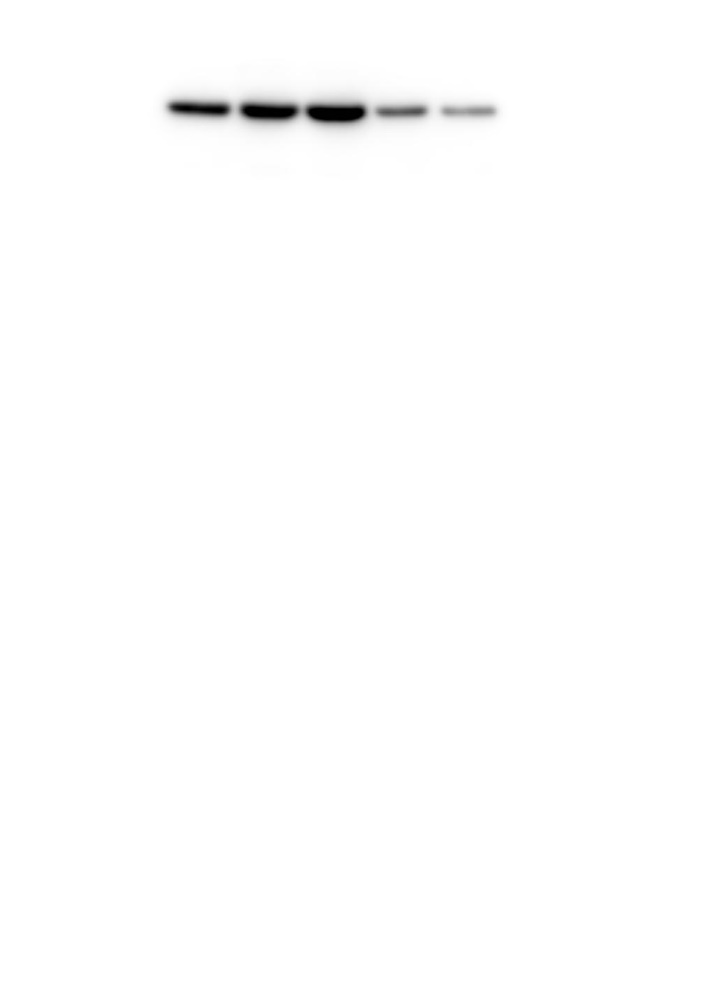


ACTB


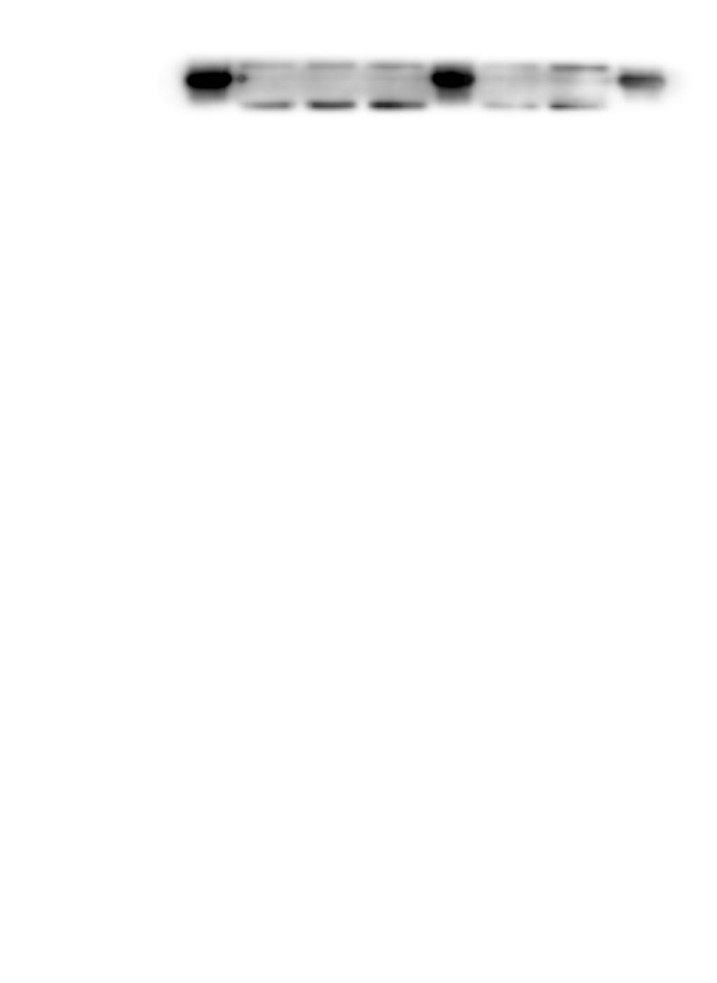


P62

**Fig. S3A
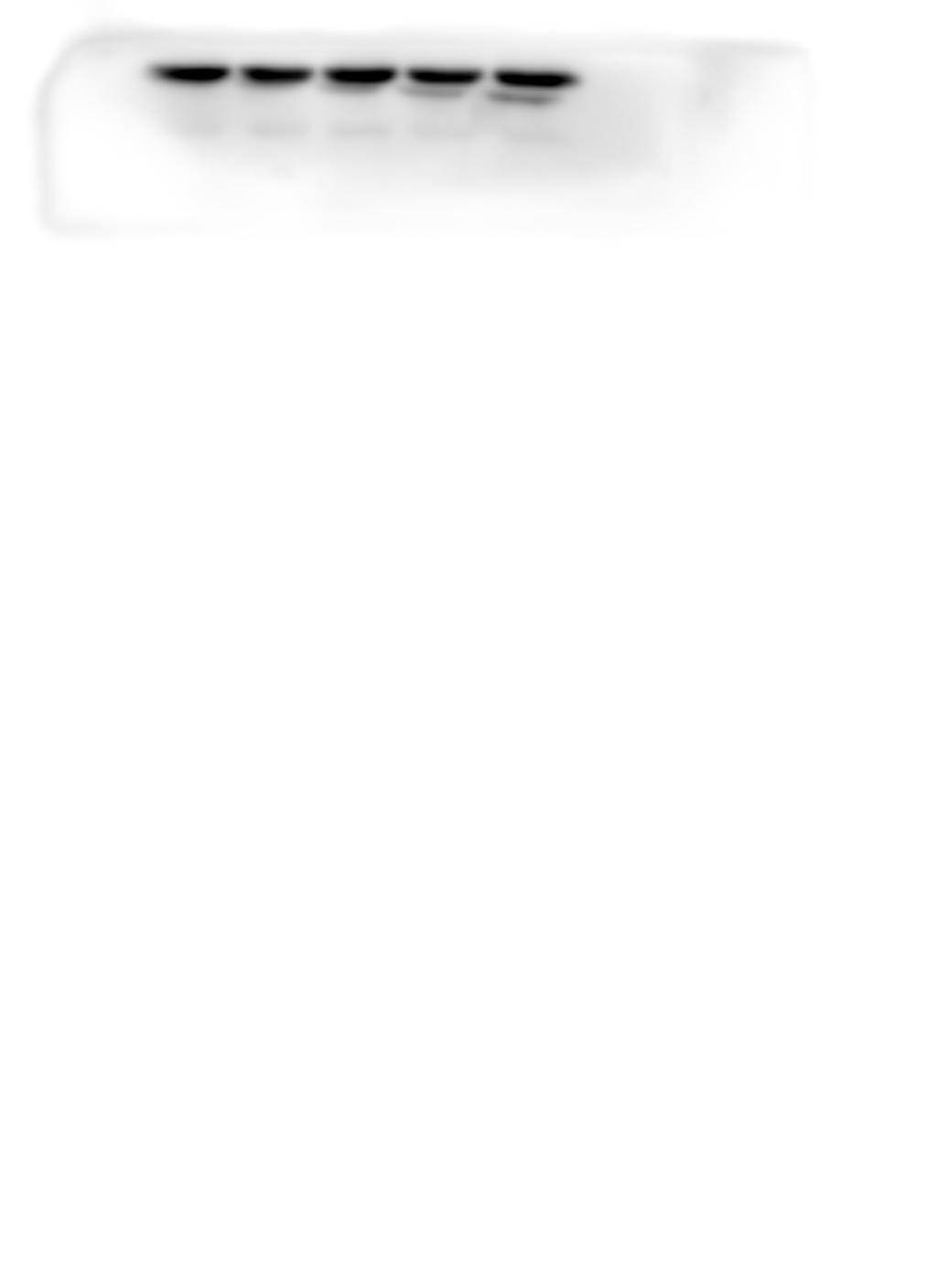
**


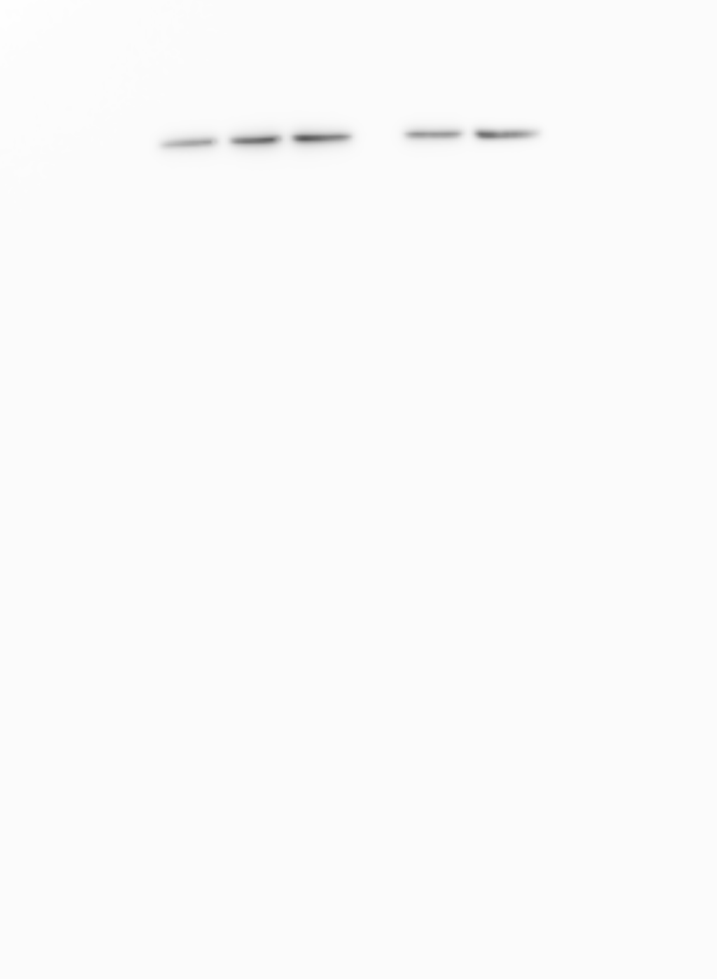


LC3I

LC3II


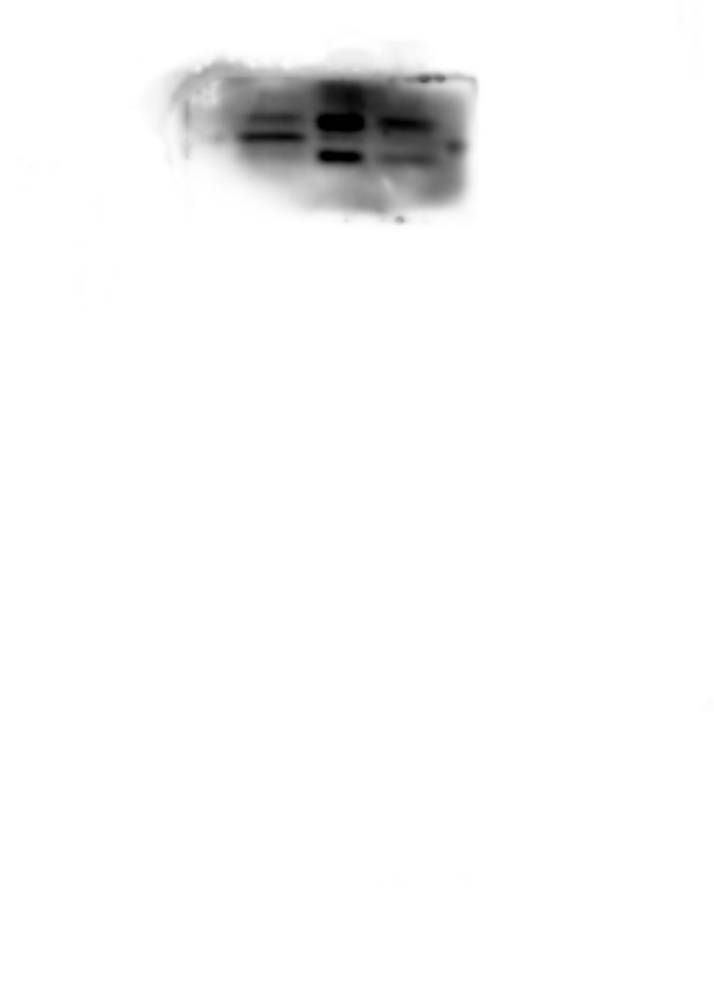


ACTB


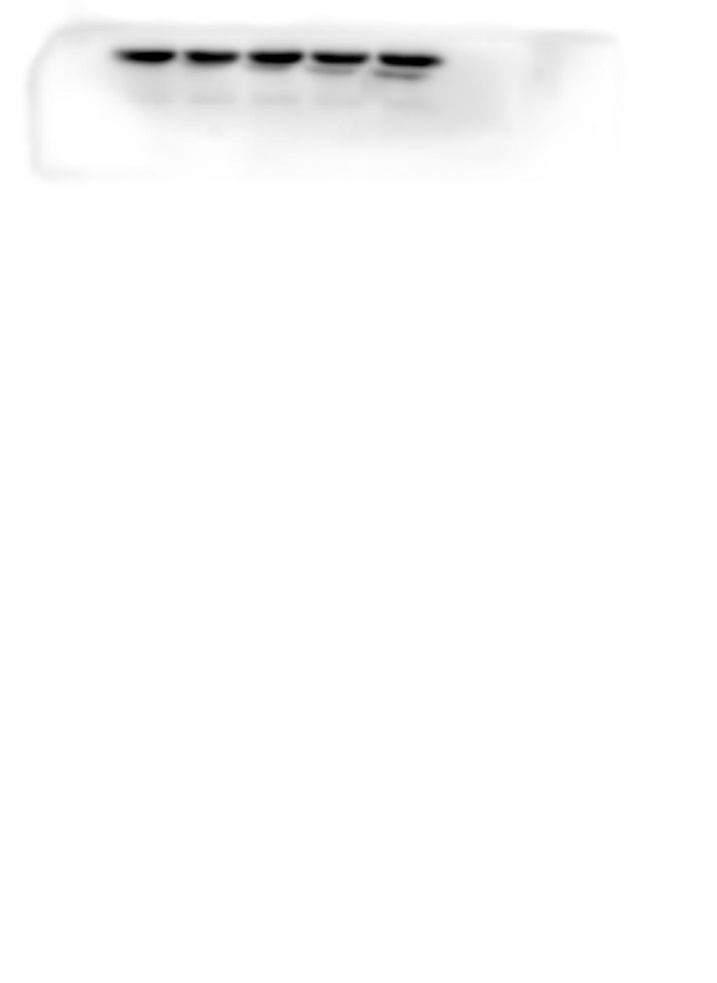

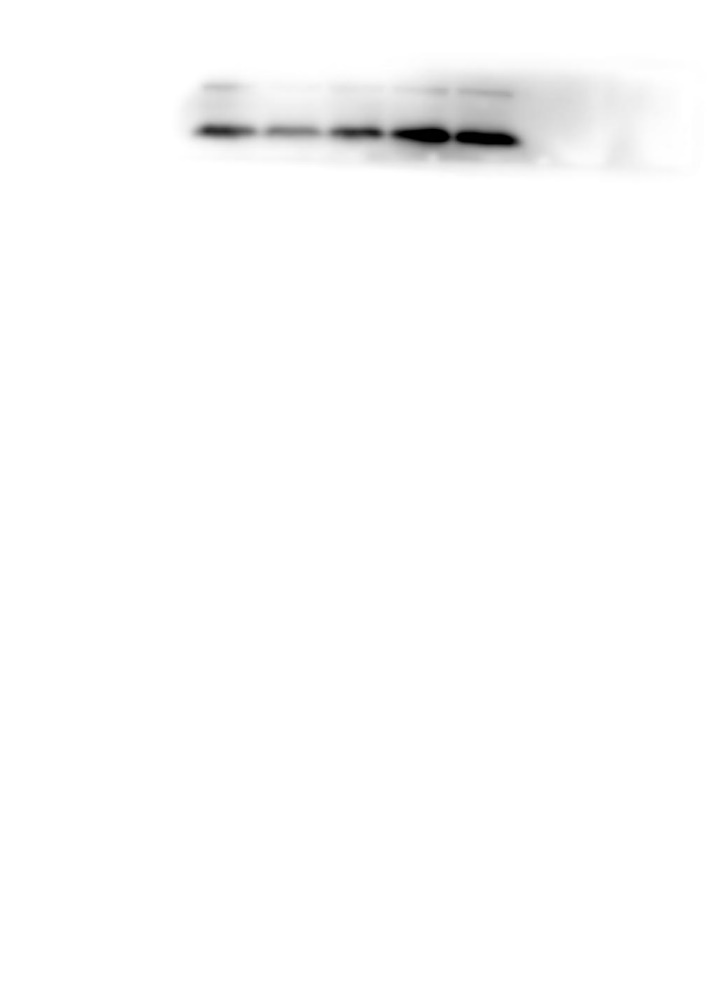

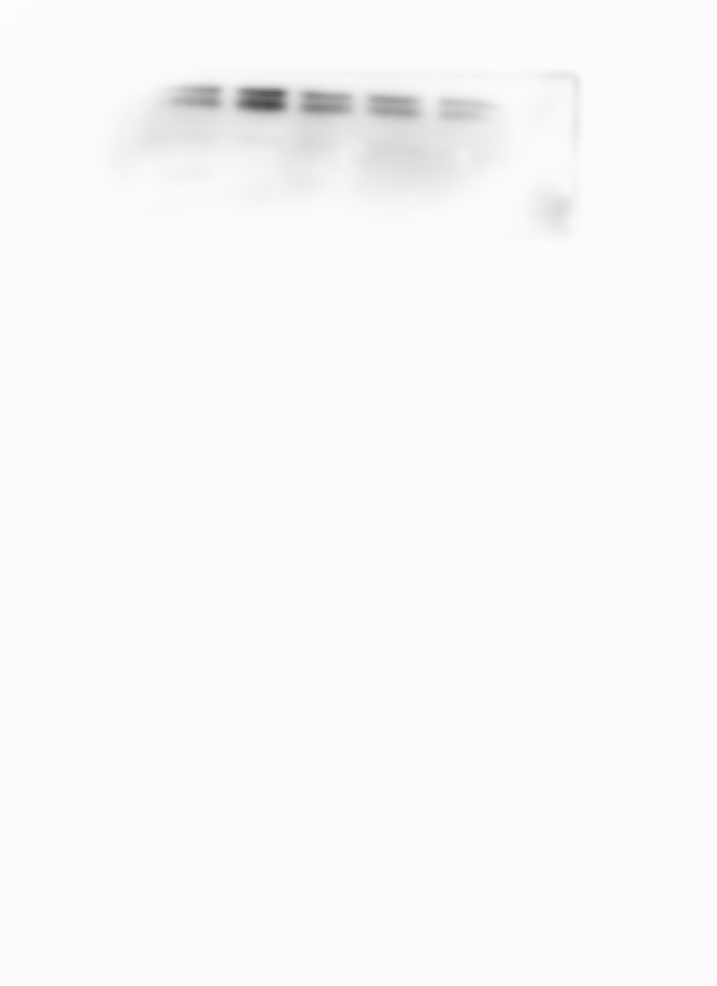


P62

LC3I

LC3II

ACTB

**Fig. S1F
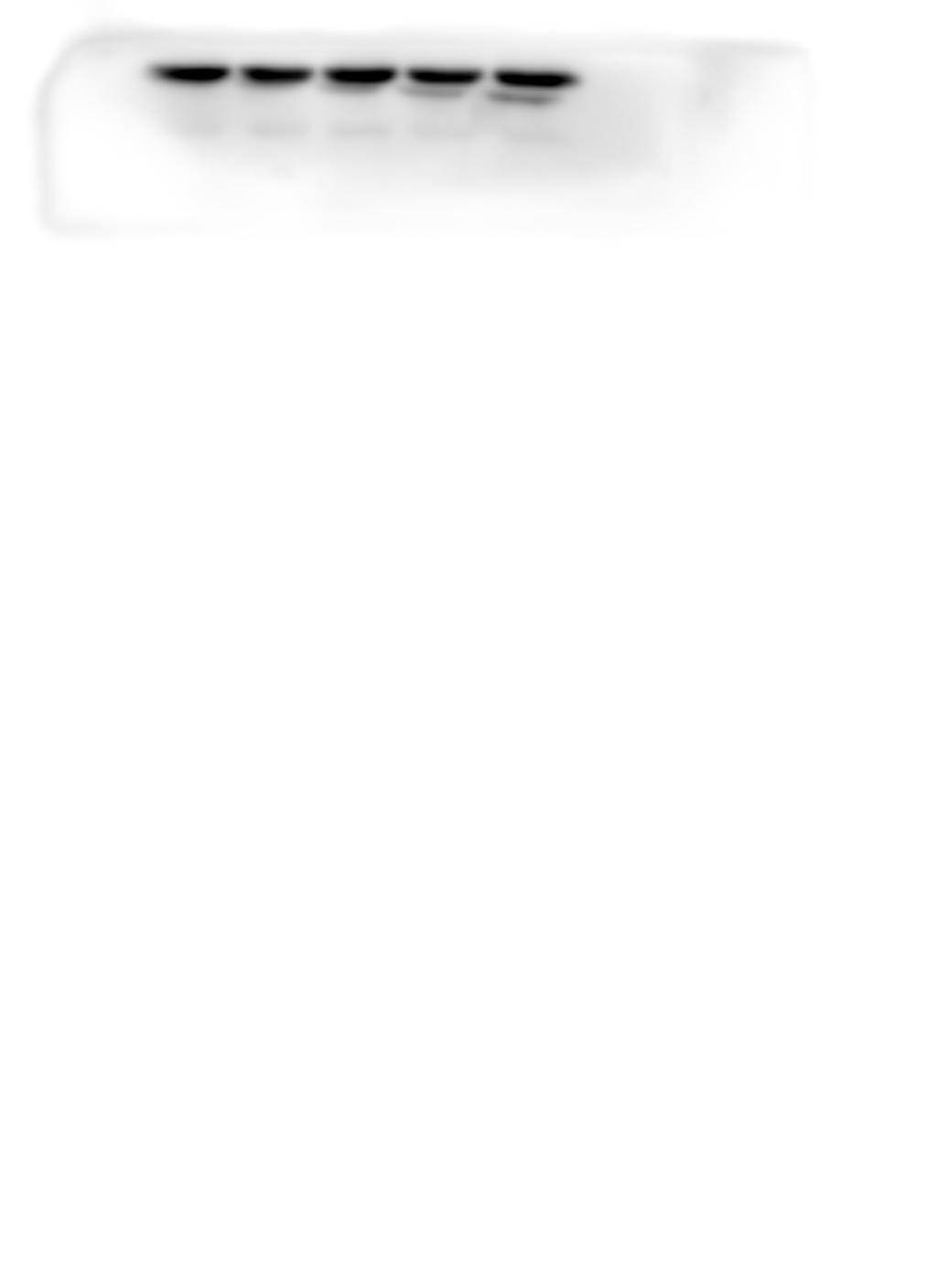
**
