# Supplementary material for: MGCG regulates glioblastoma tumorigenicity via hnRNPK/ATG2A and promotes autophagy
Source: Cell Death Dis. 2023 Jul 17;14(7):443. doi: 10.1038/s41419-023-05959-x (PMC10352271; doi:10.1038/s41419-023-05959-x)
Supplement: Supplementary file 3 — Supplementary information [file 41419_2023_5959_MOESM3_ESM.pdf]

Inventory of supplementary information

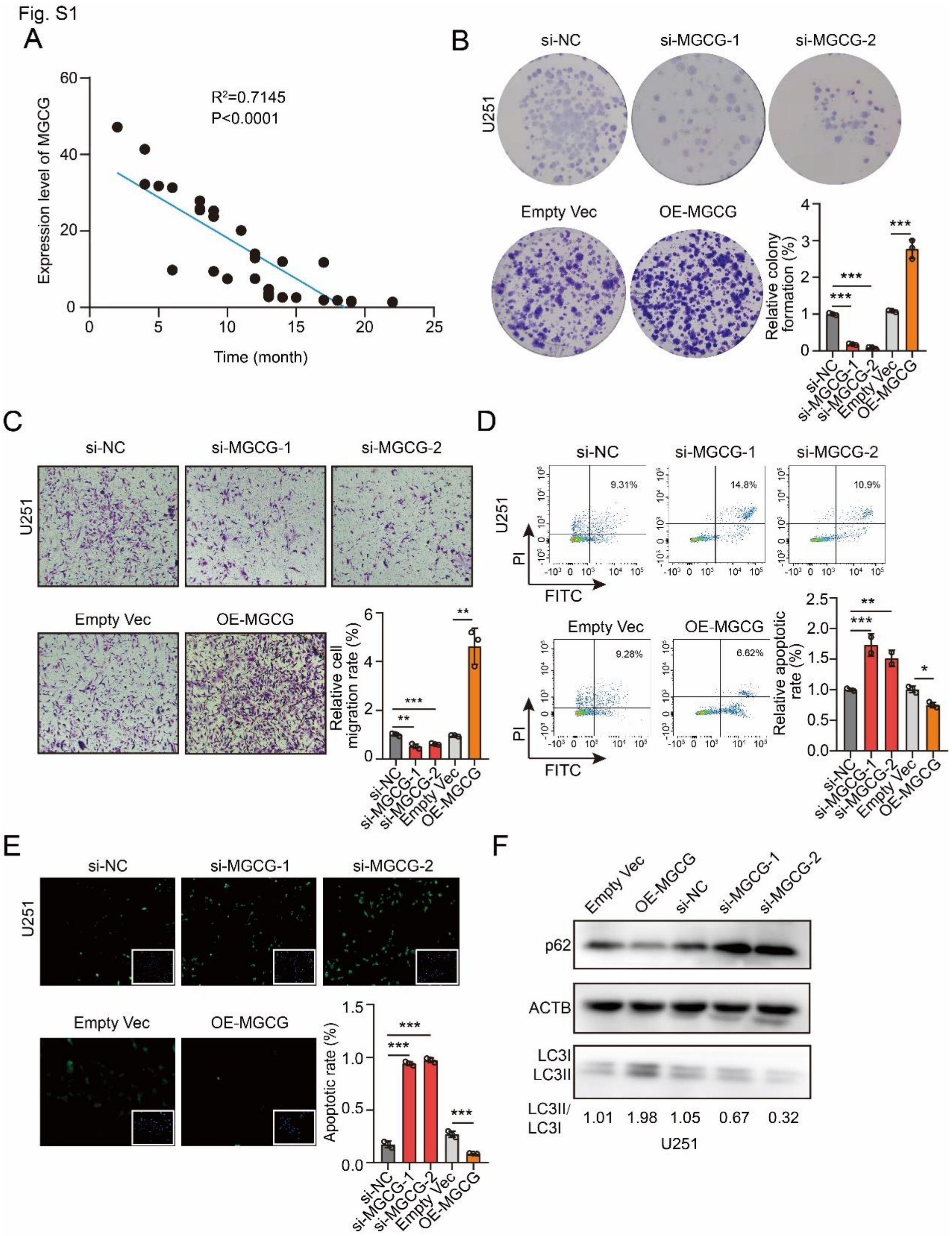

Fig. S1 MGCG promotes GBM progression *in vitro*.

A Correlation analysis of MGCG expression level and survival time in 34 GBM patients.

B Detection of the effect of MGCG knockdown or overexpression on the growth of U251 cells by a colony formation assay. **C** The data of Transwell analysis show the effect of

MGCG knockdown or overexpression on the migration of U251 cells. **D** The data of Flow cytometry analysis reveal the effect of MGCG knockdown or overexpression on apoptosis of U251 cells. **E** The data of TUNEL analysis show the effect of MGCG knockdown or overexpression on apoptosis of U251 cells. **F** Western blot analysis of the protein levels of p62 and LC3 after knockdown or overexpression of MGCG in U251 cells.

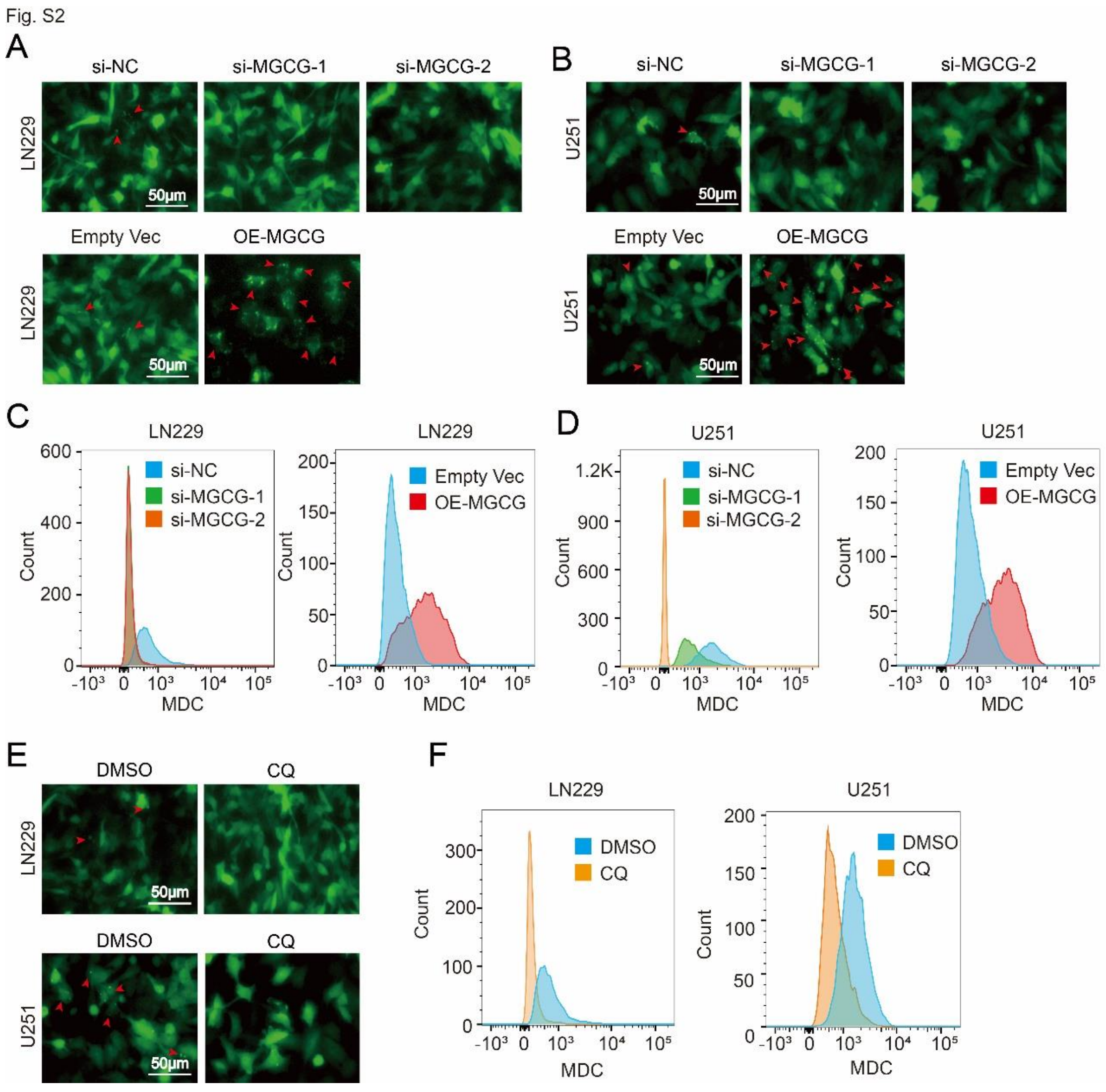

Fig. S2 MGCG facilitates autophagy in GBM cells.

A GFP-LC3 adenovirus infected LN229 cells with MGCG knockdown or overexpression to

detect autophagy. Red arrow: autophagosome. **B** GFP-LC3 adenovirus infected U251 cells with MGCG knockdown or overexpression to detect autophagy. Red arrow: autophagosome. **C** Detection of autophagy of MGCG knockdown or overexpression by MDC stain and flow cytometry in LN229 cells. MDC: monodansylcadaverin. **D** Detection of autophagy of MGCG knockdown or overexpression by MDC stain and flow cytometry in U251 cells. **E** LN229 and U251 cells were treated with DMSO and CQ, GFP-LC3 adenovirus infected the LN229 and U251 cells to detect autophagy. Red arrow: autophagosome. CQ: chloroquine. **F** LN229 and U251 cells were treated with DMSO and CQ, detection of autophagy by MDC stain and flow cytometry.

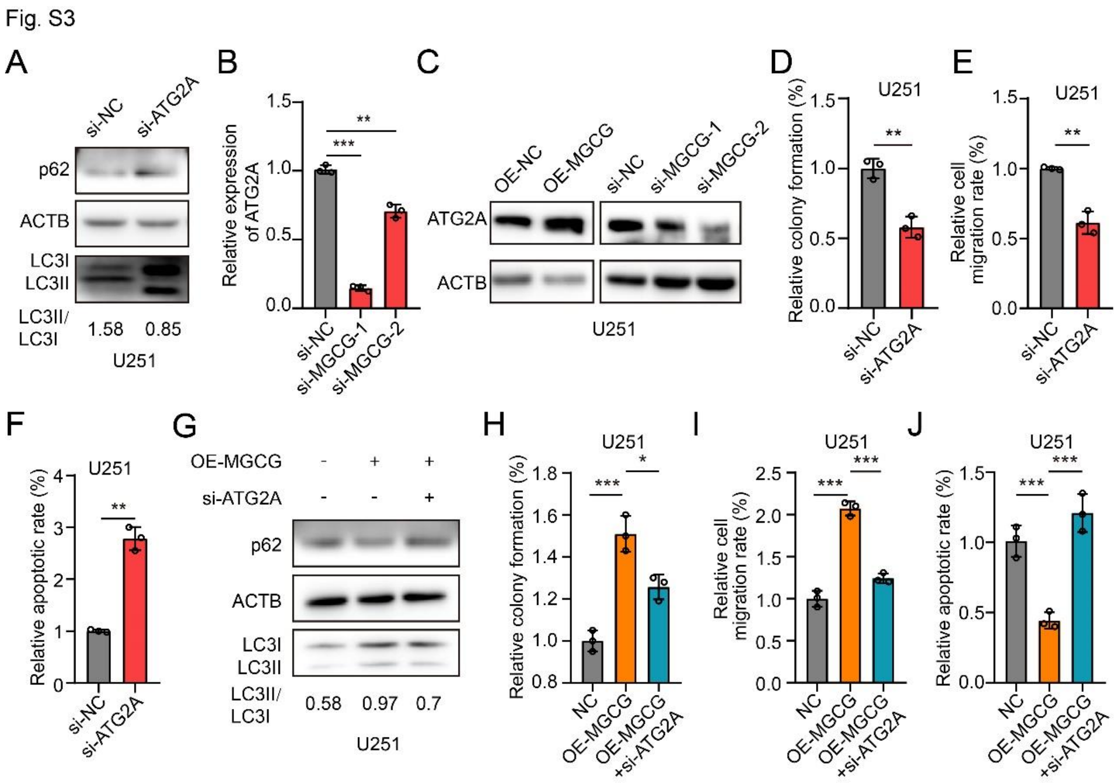

Fig. S3 MGCG facilitates tumor progression by promoting the expression of ATG2A in U251 cells.

A Western blot analysis was used to detect the protein levels of p62 and LC3 after ATG2A

knockdown in U251 cells. **B** RT–qPCR analysis of the relative expression of ATG2A after MGCG knockdown in U251 cells. **C** Western blot analysis of the protein levels of ATG2A after MGCG overexpression or knockdown in U251 cells. **D** Colony formation assays were used to detect the effect of ATG2A knockdown on the growth of U251 cells. **E** The data of Transwell analysis show the effect of ATG2A knockdown on the migration of U251 cells. **F** The data of TUNEL analysis shows the effect of ATG2A knockdown on apoptosis of U251 cells. **G** Western blotting was used to detect the protein levels of p62 and LC3 after overexpression of MGCG in U251 cells. **H** Colony formation assays were used to detect the effect of MGCG overexpression or overexpression and simultaneous knockdown of ATG2A on the growth of U251 cells. **I** The data of Transwell analysis show the effect of MGCG overexpression or overexpression and simultaneous knockdown of ATG2A on the migration of U251 cells. **J** The data of TUNEL analysis show the effect of MGCG overexpression or overexpression of MGCG and simultaneous knockdown of ATG2A on apoptosis of U251 cells. Error bars, S.E.M. from three independent experiments. *$P < 0.05$; **$P < 0.01$; ***$P < 0.001$ by two-tailed Student's test.
