# Supplementary material for: MGCG regulates glioblastoma tumorigenicity via hnRNPK/ATG2A and promotes autophagy
Source: Cell Death Dis. 2023 Jul 17;14(7):443. doi: 10.1038/s41419-023-05959-x (PMC10352271; doi:10.1038/s41419-023-05959-x)
Supplement: Supplementary file 4 — Table S1 [file 41419_2023_5959_MOESM4_ESM.docx]

**Supplementary Table 1 Oligos used in the study**

| MGCG-F | TGTGGCGGAGTTGCAAGCAA | For MGCG  qPCR |
| --- | --- | --- |
| MGCG-R | CGTGCATGACGCCACTATGA |  |
| GAPDH-F | CTTCATTGACCTCAACTACATGG | For GAPDH  qPCR |
| GAPDH-R | CTCGCTCCTGGAAGATGGTGAT |  |
| hnRNPK-F | CAATGGTGAATTTGGTAAACGCC | For hnRNPK  qPCR |
| hnRNPK-R | GTAGTCTGTACGGAGAGCCTTA |  |
| ATG2A-F | GCTCAGGGTACATGGAGCTG | For ATG2A  qPCR |
| ATG2A-R | CTCGTGGTCTGTAAGGCTCAC |  |
| U6-F | GGGGACATCCGATAAAATTG | For U6  qPCR |
| U6-R | GGACCATTTCTCGATTTGTG |  |
| DNMT1-F | GCGGCTCAAAGATTTGGAAAGA | For DNMT1  qPCR |
| DNMT1-R | CAGGTAGCCCTCCTCGGAT |  |
| DNMT3A-F | CCGATGCTGGGGACAAGAAT | For DNMT3B  qPCR |
| DNMT3A-R | CCCGTCATCCACCAAGACAC |  |
| DNMT3B-F | AGGGAAGACTCGATCCTCGTC | For DNMT3B  qPCR |
| DNMT3B-R | GTGTGTAGCTTAGCAGACTGG |  |
| Left M primer | GTAGGTGGGTAGTGGTTACGATAAC | MSP |
| Right M primer | CTTCCCTCAATACCTAATACAACGA |  |
| Left U primer | TGTAGGTGGGTAGTGGTTATGATAAT |  |
| Right U primer | CCTTCCCTCAATACCTAATACAACA |  |
| si-MGCG-1 | CUCAUCUUUGUUAUUUGUUCA  AACAAAUAACAAAGAUGAGAG | siRNAs of MGCG |
| si-MGCG-2 | AACAAAUAACAAAGAUGAGAG  CUCAUCUUUGUUAUUUGUUCA |  |
| si-MGCG-3 | AGGAAAUAUCUUUGGAAUGUA  CAUUCCAAAGAUAUUUCCUUU |  |
| si-hnRNPK | AAUCAGUUAUUAUAUAUCCUU  GGAUAUAUAAUAACUGAUUGG | siRNAs of hnRNPK |
| si-ATG2A | CACTTTTTCACCGAGTTTGATGC  GACCTACATGGTATCTATGAAGA | siRNAs of ATG2A |
| si-NC | UUCUCCGAACGUGUCACGU  ACGUGACACGUUCGGAGAA | Negative control |
| MGCG-probe1 | CGGGATGCGACTTATGATGGCTTGGCGGAT | For MGCG RIP with 5’biotin labeled |
| MGCG-probe2 | AAAATAATAGGCATCACCCCAATAGGCAAT |  |
| MGCG-probe3 | AATATTATTTTTCTTGAACCCTTTCAGCGC |  |
| ATG2A-probe1 | ATTGGCAACGGGCAGGCTGGGAAGGCGTCC | For ATG2A RIP with 5’biotin labeled |
| ATG2A-probe2 | GCCGCCTGGGCTTGCCGCCCGCCGGCGATC |  |
| Scramble | TTCTCCGAACGTGTCACGTTCGAACGTGTC | Control probe with 5’biotin labeled |
